# BODIPY-Decorated Nanoscale Covalent Organic Frameworks for Photodynamic Therapy

**DOI:** 10.1016/j.isci.2019.03.028

**Published:** 2019-03-29

**Authors:** Qun Guan, Dan-Dan Fu, Yan-An Li, Xiang-Mei Kong, Zhi-Yuan Wei, Wen-Yan Li, Shao-Jun Zhang, Yu-Bin Dong

**Affiliations:** 1 College of Chemistry, Chemical Engineering and Materials Science, Collaborative Innovation Center of Functionalized Probes for Chemical Imaging in Universities of Shandong, Key Laboratory of Molecular and Nano Probes, Ministry of Education, Shandong Normal University, Jinan 250014, P. R. China; 2 Qianfoshan Hospital of Shandong Province, Jinan 250014, P. R. China; 3 Binzhou Medical University (Yantai Campus), Yantai 264003, P. R. China

**Keywords:** Supramolecular Chemistry, Scaffolds in Supramolecular Chemistry, Cancer, Biomedical Materials

## Abstract

Covalent organic frameworks (COFs), an emerging class of organic porous materials, have attracted intense attention due to their versatile applications. However, the deliberate fabrication of COF-based nanomaterials for nanomedical application remains challenging due to difficulty in their size- and structure-controlled synthesis and poor aqueous dispersibility. Herein, we report two boron-dipyrromethene (BODIPY)-decorated nanoscale COFs (NCOFs), which were prepared by the Schiff-base condensation of the free end –CHO (bonding defects in COFs) on the established imine-based NCOFs with the amino-substituted organic photosensitizer BODIPY via “bonding defects functionalization” approach. Thus BODIPY has been successfully nanocrystallized via the NCOF platform, and can be used for photodynamic therapy (PDT) to treat tumors. These NCOF-based PDT agents featured nanometer size (∼110 nm), low dark toxicity, and high phototoxicity as evidenced by *in vitro* and *in vivo* experiments. Moreover, the “bonding defects functionalization” approach might open up new avenues for the fabrication of additional COF-based platforms for biomedical treatment.

## Highlights

• Covalent organic frameworks (COFs) are first used in tumor photodynamic therapy
• BODIPY-decorated COFs are synthesized via bonding defects functionalization
• BODIPY-decorated COFs have excellent anti-tumor efficacy *in vitro* and *in vivo*
• COFs show great promise as nanoplatforms for biomedical applications

## Graphical Abstract

**Figure:**
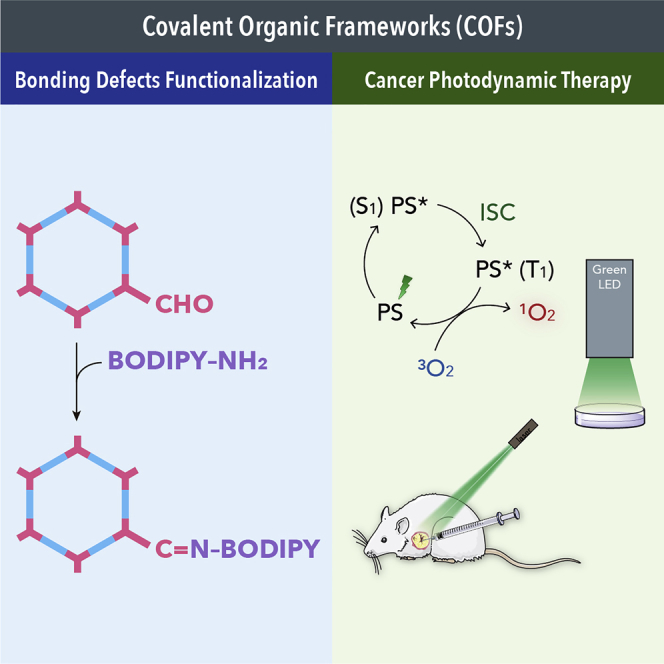

## Introduction

As is known, cancer is one of the greatest threats to human health (Fitzmaurice et al., 2018, Siegel et al., 2019). Photodynamic therapy (PDT) (Bolze et al., 2017, Guan et al., 2018a, Mallidi et al., 2016) is a promising clinical cancer treatment method in which a light-absorbing agent, referred to as a *photosensitizer* (PS), is interacted with light and oxygen to produce cytotoxic singlet oxygen (^1^O_2_). Compared with chemotherapy and radiotherapy, PDT generates less collateral damage to normal tissues, because ^1^O_2_ is produced only in the illuminated area where the PS accumulates. In this context, some organic dyes, such as boron-dipyrromethene (BODIPY)- and porphyrin-based species (Bertrand et al., 2018, Durantini et al., 2018, Josefsen and Boyle, 2012, Rajora et al., 2017), have been demonstrated to be the highly effective PSs owing to their high extinction coefficient and low dark toxicity in this minimally invasive cancer treatment. Their practical application, however, is often limited by the poor water solubility and instability, aggregation, sometimes photobleaching, and low cell permeability (Li et al., 2018a, Zhou et al., 2018).

Organic PS nanocrystallization has been proved to be an alternative approach to address the aforementioned issues (Abánades Lázaro and Forgan, 2019, Lismont et al., 2017). For example, recent studies revealed that organic PSs can be readily nanocrystallized via nanoscale metal-organic framework (NMOF) platform by either one-pot (Guan et al., 2018b, Lu et al., 2014) or post-synthetic modification (PSM) (Kan et al., 2018, Nian et al., 2017, Wang et al., 2016). In such a way, the molecular organic PSs are encapsulated in NMOF pores or are covalently attached to the established NMOF frameworks, and their aggregation and self-quenching are therefore effectively avoided due to their periodic arrangement within the MOF framework (Qin et al., 2019). In addition, nanocrystallization of the organic PSs significantly improves their endocytosis-based cellular uptake (Mosquera et al., 2018, Yoon and Rossi, 2018), and consequently, augments their cancer therapeutic efficacy.

As a promising alternative to MOFs, *metal-free* covalent organic frameworks (COFs) (Bisbey and Dichtel, 2017, Lohse and Bein, 2018) should be more biocompatible and suitable for biomedical treatment because of their pure organic nature (Figure S1, see Normal Tissue Cytotoxicity Test in Transparent Methods for experimental details) (Guan et al., 2018b, Jiang et al., 2019, Ruyra et al., 2015, Tamames-Tabar et al., 2014). Some examples of COF-based biomedical applications have been reported, such as fluorescence bioimaging (Das et al., 2018, Li et al., 2017, Liu et al., 2019b, Wang et al., 2018a), drug delivery (Bai et al., 2016, Fang et al., 2015, Liu et al., 2019a, Mitra et al., 2017, Vyas et al., 2016b, Zhang et al., 2018b), antibacterial therapy (Hynek et al., 2018, Liu et al., 2017, Mitra et al., 2016), enzyme immobilization (Kandambeth et al., 2015, Sun et al., 2018c), biochemical analysis (Liu et al., 2019c, Yan et al., 2019, Zhang et al., 2018e, Zhang et al., 2018f, Zhou et al., 2019), and so on. In addition, some conceptual therapeutic models, such as ^1^O_2_ generation (Feng et al., 2016, Lin et al., 2017, Nagai et al., 2013), photothermal conversion (Tan et al., 2016), and apoptosis induction (Bhanja et al., 2017), have also been reported. To the best of our knowledge, nanoscale COF (NCOF)-based PSs for tumor PDT, have not been reported so far, although they are conceptually practicable. This might be limited by the following issues: (1) COFs are usually obtained as microscale particles with low aqueous dispersibility, which are not conducive to cell uptake; (2) *in situ* one-pot synthesis of NCOF-based PSs from PS-attached building blocks might not be versatile because it is difficult to ensure that the PSs are intact during COF synthesis; (3) covalent decoration of the molecular PSs on the established COF framework by the existing PSM approach might also be limited by the stability and activity of the pre-embedded active precursors under the harsh COF synthetic conditions, and moreover, the incoming bulky PS molecules probably lead to crystallinity decrease, structural transformation, and even structure collapse of the pristine COFs.

Based on recent reports, NCOF synthesis can be realized by the polymer-assisted solvothermal method (Zhao et al., 2017) or with the help of ultrasonic stripping (Chandra et al., 2013, Zhang et al., 2017a). On the other hand, COFs, which are composed of organic building partners via the covalent bonds, contain the unbonded functional groups (bonding defects) at the end of COF matrix (Nguyen and Grünwald, 2018), which should be a heaven-sent opportunity to graft small organic PSs onto the established NCOF with a known structure via bonding defects functionalization (BDF). In this way, the isostructural but PS-attached NCOFs that cannot be directly prepared by the conventional one-pot synthesis and existing PSM would be readily achieved.

**Figure 1:**
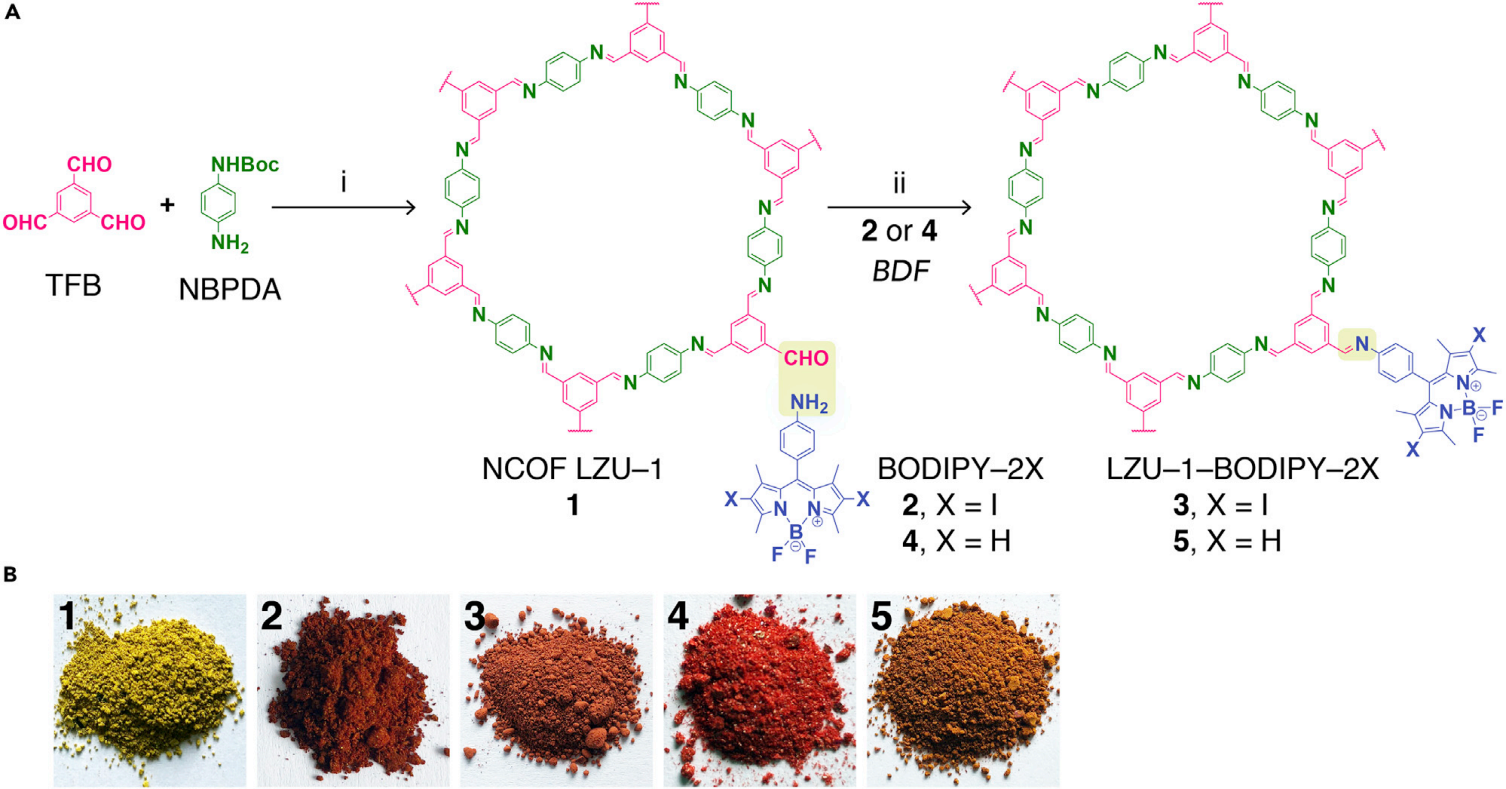
Design of BODIPY-Decorated Nanoscale COFs for PDT (A) The preparation of **1**, **3**, and **5**. (i) PVP, EtOH, CF_3_COOH, 120°C, 12 h; (ii) EtOH, HOAc, 75°C, 4 h. Boc, *tert*-butyloxycarbonyl; BDF, bonding defects functionalization. See Synthesis of NCOF LZU-1 (**1**), Synthesis of BODIPY-2I (**2**), Synthesis of LZU-1-BODIPY-2I (**3**), Synthesis of BODIPY-2H (**4**), and Synthesis of LZU-1-BODIPY (**5**) in Transparent Methods for experimental details. (B) Digital photographs of **1**, **2**, **3**, **4**, and **5**.

In this contribution, we report, the first of its kind, two BODIPY-decorated NCOFs, which were generated from the NCOF LZU-1 (**1**) (Ding et al., 2011) and two amino-decorated BODIPY molecules, termed *BODIPY-2I* (**2**) and *BODIPY-2H* (**4**), by the BDF approach under the given conditions (Figure 1A). Markedly, the obtained nanoscale LZU-1-BODIPY-2I (**3**) and LZU-1-BODIPY-2H (**5**) are highly crystalline and isostructural to pristine **1**, and they both feature low cytotoxicity, good biocompatibility, high cancer cell uptake, and highly efficient ^1^O_2_ generation. Therefore, they can be used as high-performing PDT agents for cancer treatment under the given *in vitro* and *in vivo* conditions.

## Results

### Synthesis and Characterization

With the aid of nonionic surfactant polyvinyl pyrrolidone, the NCOF of LZU-1 (**1**) was prepared by the combination of benzene-1,3,5-tricarbaldehyde and *tert*-butyl (4-aminophenyl)carbamate (NBPDA) under solvothermal conditions (EtOH, CF_3_COOH, 120°C, 12 h) (Zhao et al., 2017). Furthermore, the BDF of **1** via Schiff-base condensation between its free end aldehyde groups and two amino-decorated BODIPY molecules, termed as *BODIPY-2I* (**2**) and *BODIPY-2H* (**4**), afforded nanoscale LZU-1-BODIPY-2I (**3**) and LZU-1-BODIPY-2H (**5**) under solvothermal conditions (EtOH, HOAc, 75°C, 4 h) (Figure 1A, see Synthesis of NCOF LZU-1 (**1**), Synthesis of BODIPY-2I (**2**), Synthesis of LZU-1-BODIPY-2I (**3**), Synthesis of BODIPY-2H (**4**), and Synthesis of LZU-1-BODIPY (**5**) in Transparent Methods for experimental details). The colors of **3** and **5** were significantly different from those of their precursors after the reaction (Figure 1B). The standard curve method (Figure S2, see BODIPY Contents Determination in Transparent Methods for experimental details) revealed decorated BODIPY amounts in **3** and **5** of 0.1360 ± 0.0312 mmol/g and 0.1545 ± 0.0220 mmol/g, respectively. This result was further confirmed by inductive coupled plasma optical emission spectrometry (ICP-OES) measurement (0.1218 ± 0.0137 mmol/g, 0.1492 ± 0.0278 mmol/g, respectively). Owing to their structural similarity, we took **3** as an example to discuss its structural characterization in detail herein, and the corresponding detailed characterization data for **5** are provided in Figures S3 and S4.

**Figure 2:**
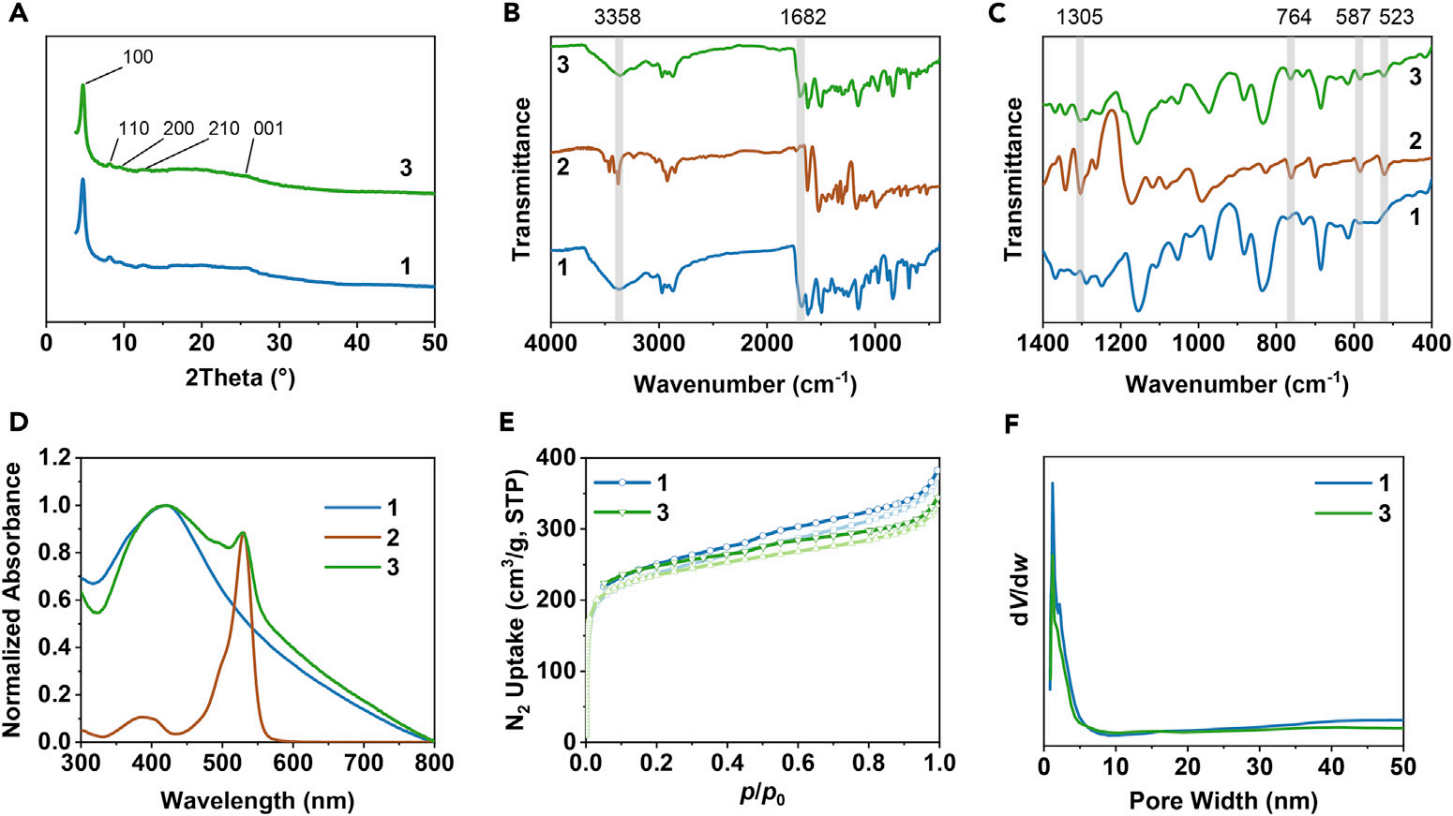
Spectroscopic Characterization of **1** and **3** (A) PXRD patterns of **1** and **3**. (B and C) Fourier transform infrared spectra (B) and their fingerprint regions (C) of **1**, **2**, and **3**. (D) UV-vis absorption spectra of **1**, **2**, and **3** in DMF. (E) N_2_ adsorption-desorption isotherms (77 K) of **1** and **3**. (F) Pore width distribution plots of **1** and **3**.

The obtained **1** and **3** are isostructural and possess good crystallinity, as revealed by their experimental powder X-ray diffraction (PXRD). As indicated in Figure 2A, the most intense peak at 2*θ* = 4.7° was attributed to the (100) crystal facet, and other diffraction peaks at 2*θ* = 8.2, 9.5, and 12.4° were assigned to the (110), (200), and (210) facets, respectively. The broad peak at 2*θ* = 25.7° could be indexed to the π–π stacked planes (001) of **1** (Peng et al., 2016). This suggested that the BDF of **1** with **2** did not change its structural integrality and crystallinity under the given conditions.

The successful formation of **1** and **3** was assessed by Fourier transform infrared spectroscopy. As shown in Figure 2B, the strong stretching band of C=N in **1** appeared at 1,621 cm^−1^, clearly indicating the formation of the imine linkage (Yuan et al., 2018). Meanwhile, the peaks at 1,682 and 3,358 cm^−1^ associated with aldehyde and amino groups, respectively, in **1** significantly decreased, but did not completely disappear, suggesting the existence of bonding defects. After BDF, the residual peak for the aldehyde group still existed, which demonstrated the BDF between **1** and **2** was not quantitative, which might result from the inherent imperfection of the heterogeneous solid-liquid reaction. Notably, as shown in Figure 2C, the characteristic peaks of **2** at 1,305, 764, 587, and 523 cm^−1^ were found in the fingerprint region of **3**, which could be direct evidence for the existence of BODIPY species in **3**. The formation of **1** and **3** was further confirmed by ^13^C solid-state nuclear magnetic resonance (Figure S4). It is worth noting that the changes observed for the resonance signals at *δ* = 17.3 ppm for **3** unequivocally supported the existence of BODIPY species.

The ultraviolet-visible (UV-vis) absorption spectra of **1**, **2**, and **3** were recorded in *N*,*N*-dimethylformamide (DMF). As shown in Figure 2D, **3** displayed the characteristic absorption bands of both **1** and **2**. A broadband absorption of **1** and **3** throughout the whole visible light region was observed, indicating their stacked layer structures with enhanced light-harvesting capability in a wide range of visible light region due to the delocalized π electrons in the COFs. Compared with **2**, the broadened characteristic absorption peak of BODIPY at 530 nm in **3** was also observed but without band-shift, indicating that the involved BODIPY species in **3** were well dispersed, as we know that aggregated BODIPY would cause the adsorption band to be significantly red-shifted (Li et al., 2018d, Liu et al., 2019d, Zhang et al., 2016). More importantly, this observation demonstrated that **3** is a COF-BODIPY covalently bonded species rather than a simply physical blend or a host-guest complex.

To examine the permanent porosity, N_2_ adsorption properties of **1** and **3** were measured at 77 K. As shown in Figure 2E, the Brunauer-Emmett-Teller (BET) surface areas of **1** and **3** were 822 m^3^/g and 805 m^3^/g, respectively. The slightly decreased surface area in **3** should be caused by the introduced BODIPY species because the involved BODIPY species certainly enhanced the material weight, thus resulting in a corresponding decrease of d*V*/d*w*. Density functional theory fitting of the adsorption branches (Figure 2F) showed the average pore size distributions of both **1** and **3** centered at ca. 1.2 nm, implying that the covalently bonded BODIPY species should be located at the end of the COF matrix.

To further verify the covalent bonding of BODIPY species of **2** and **4** to the COF framework of **1**, a control experiment was designed and conducted (Figure S5, see Control Experiments in Transparent Methods for experimental details). When **1** was separately impregnated in an EtOH solution of **2** and **4** for 4 h, the host-guest complexes of BODIPY-2I⊂LZU-1 (**3′**) and BODIPY-2H⊂LZU-1 (**5′**) were generated. As determined by the standard curve method, the loading amounts of BODIPY in **3′** and **5′** were up to 0.2149 ± 0.0875 mmol/g and 0.5130 ± 0.1763 mmol/g, respectively, which are significantly higher than those of **3** and **5**. It is different from the case of **3** and **5;** the BODIPY loading amount herein is size dependent, and smaller-sized **4** (9 Å) was more uploaded than its diiodio-substituted analog of **2** (11 Å), which is a typical phenomenon in the selective host-guest adsorption. In addition, the release kinetics of **3**, **3′**, **5**, and **5′** were investigated in boiling ethanol. As expected, the encapsulated BODIPY in **3′** and **5′** was readily extracted with EtOH with a rapid BODIPY release. In contrast, no BODIPY leaching occurred in **3** and **5** under the same conditions because they were firmly anchored by the covalent imine bond. By BDF in this way, the nanocrystallization of BODIPY via COF platform has been successfully achieved, which laid a solid foundation for their applications in biomedical treatment.

**Figure 3:**
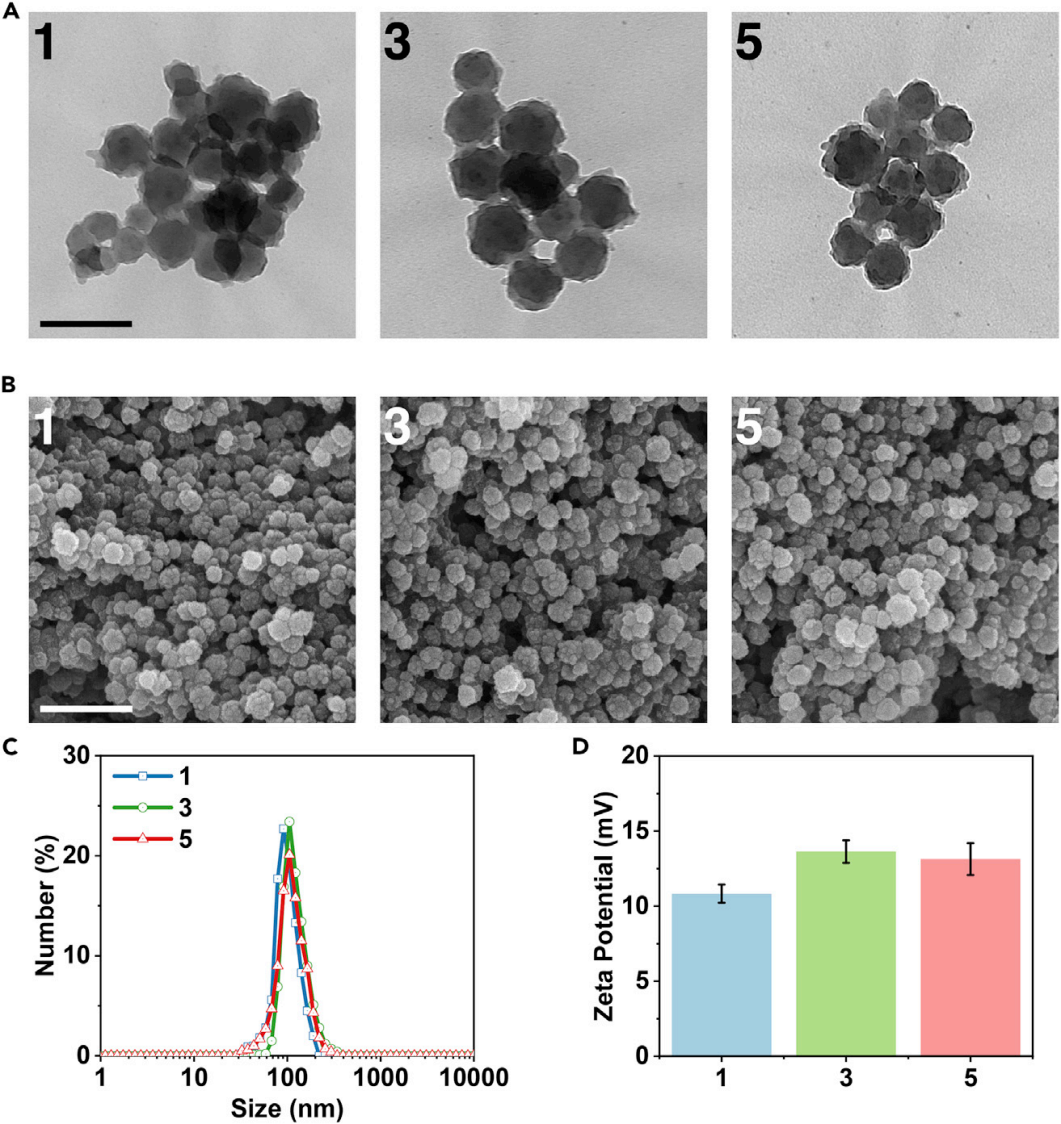
NP Microtopography Characterization (A) Transmission electron microscopic images of **1**, **3**, and **5**. Scale bar, 200 nm. (B) Scanning electron microscopic images of **1**, **3**, and **5**. Scale bar, 500 nm. (C) DLS size profiles of **1**, **3**, and **5** in PBS (pH = 6.5) at 25°C. (D) Zeta potentials of **1**, **3**, and **5** in PBS (pH = 6.5). Data are presented as mean ± SD (n = 3).

Besides structural characterization, the morphology of **1**, **3,** and **5** was investigated by scanning electron microscopy and transmission electron microscope. As indicated in Figures 3A and 3B, the obtained nanoparticles (NPs) of **1**, **3,** and **5** were uniformly distributed and their diameter was ca. 110 nm, which is further supported by the dynamic light scattering (DLS) measurement in PBS (Figure 3C). The slight difference in size might be caused by the solvation effect depending on the different measurements (Röder et al., 2017). It was worth noting that NBPDA played an important role in the synthesis of nanosized **1** (Zhao et al., 2017). With the aid of CF_3_COOH, the –Boc group could *in situ* hydrolyze and gradually release *p*-phenylenediamine under the given conditions. In this way, the Schiff condensation rate was significantly decreased, which was favorable for the formation of highly crystalline **1**. On the other hand, if *p*-phenylenediamine was used instead of NBPDA, only micron-sized LZU-1 was obtained (Figure S6). The surface chemistries of **1**, **3**, and **5** were also studied. As shown in Figure 3D, **1**, **3**, and **5** displayed positive zeta potentials in PBS (pH = 6.5), which might be caused by the protonation of N atoms in COFs. NPs with electropositive zeta potential should be of great benefit to the tumor cell uptake owing to their high affinity for the negatively charged tumor cell membranes (Chakraborty et al., 2018, Intlekofer and Finley, 2019).

### Chemical, Light, and Colloidal Stability

The stabilities of **3** and **5** were evaluated from three aspects: chemical stability in PBS, anti-bleaching ability under light irradiation, and colloidal stability of their PBS dispersions (see Chemical Stability, Light Stability, and Colloidal Stability in Transparent Methods for experimental details). After soaking in PBS (pH = 6.5) for 24 h, no change in their PXRD patterns was observed, indicating **3** and **5** featured excellent chemical stability in tumor cell microenvironment (Figure S7). In addition, there were no detectable changes in their UV-vis spectra under green laser (1 W/cm^2^) illumination for 30 min, demonstrating that **3** and **5** were not affected by external light sources, even with the high-intensity lasers (Figure S8). Moreover, when the PBS solutions of **3** and **5** were allowed to stand at room temperature after 24 h, no coagulation was observed, and the measured particle size by DLS and zeta potentials remained unchanged (Figure S9).

### Singlet Oxygen Generation

**Figure 4:**
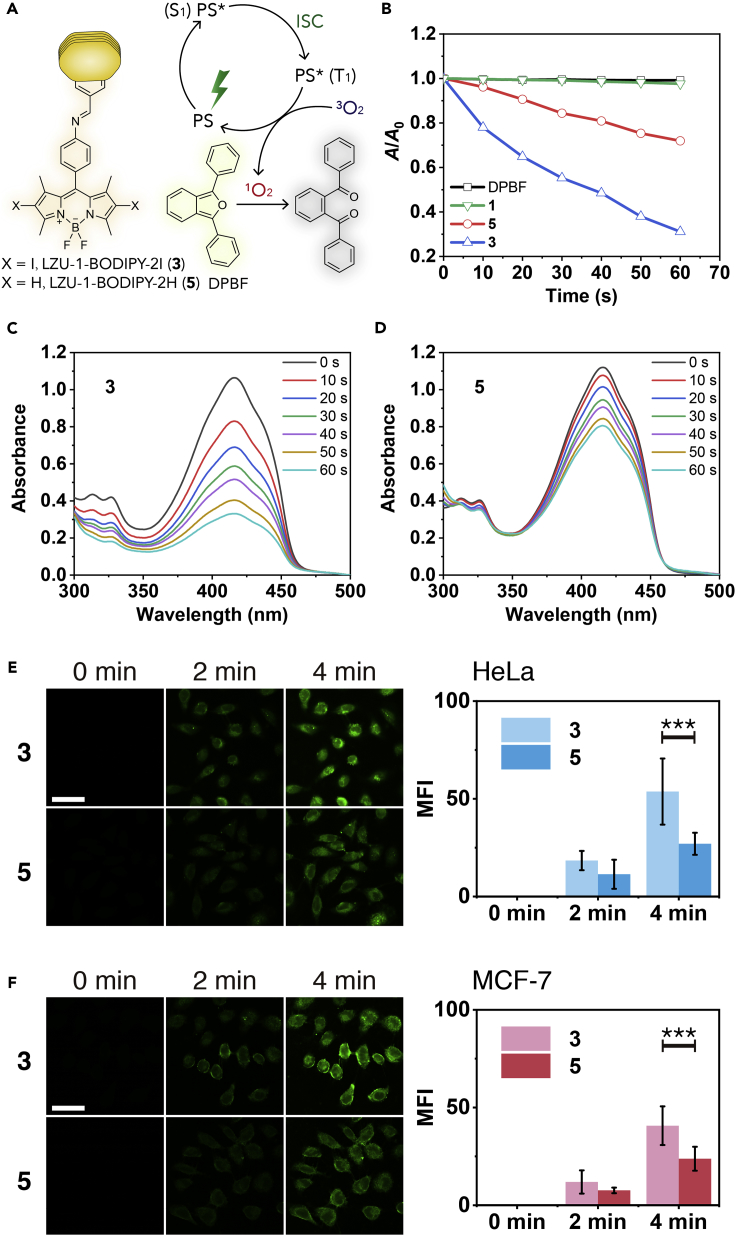
Singlet Oxygen Generation Induced by **3** and **5** (A) DPBF as a chemical probe for detecting ^1^O_2_. (B) Comparison of the decay rate of DPBF in PBS (pH = 6.5) induced by **1**, **3**, and **5** under green LED irradiation (40 mW/cm^2^). (C and D) UV-vis spectra of DPBF induced by the PBS (pH = 6.5) dispersions of (C) **3** and (D) **5** (2 mL, 10 μM, BODIPY equiv.) under green LED irradiation (40 mW/cm^2^). (E and F) HeLa (E) and MCF-7 (F) cells laser scanning confocal images of intracellular ^1^O_2_. Scale bar, 50 μm. Cells were incubated with DPBS dispersion of **3** or **5** (200 μL, 0.2 μM, BODIPY equiv.) in CO_2_ incubator for 30 min, and further incubated with SOSG (5 μM, 200 μL) for 15 min. The cells were exposed to green LED (40 mW/cm^2^) for different times and imaged with a laser scanning confocal microscope. The concentration of ^1^O_2_ in cells was reflected by mean fluorescence intensity (MFI) of green fluorescence. Data were presented as mean ± SD (n = 5, ***p < 0.001).

For PDT treatment, the ^1^O_2_ generation of **3** and **5** in PBS upon green light-emitting diode (LED) irradiation was measured by using 1,3-diphenylisobenzofuran (DPBF) (Zhang et al., 2018c) as a ^1^O_2_ probe (see Singlet Oxygen Generation in PBS in Transparent Methods for experimental details). As shown in Figures 4A–4D, the DPBF absorbance at 414 nm in the presence of **3** and **5** under green LED irradiation (40 mW/cm^2^) remarkably decreased within 1 min, implying ^1^O_2_ generation. In contrast, negligible changes were observed in control experiments (**1** + DPBF + light, and DPBF only) under the same conditions, which indicated that **3** and **5** herein were highly efficient COF-based PSs. Because intersystem crossing was significantly promoted by heavy-atom effect (Wu et al., 2011, Zou et al., 2017), **3** exhibited much higher ^1^O_2_ generation efficiency than of **5**.

As shown above, **3** and **5** exhibited excellent ability to induce ^1^O_2_ generation in PBS. To evaluate their intracellular ^1^O_2_ production ability, Singlet Oxygen Sensor Green (SOSG), which is a specific green fluorescent probe for ^1^O_2_ in cells (Jia et al., 2018), was used as a fluorescent probe to detect intracellular ^1^O_2_ generation triggered by **3** and **5** (see Intracellular Singlet Oxygen Generation in Transparent Methods for experimental details). As the irradiation time prolonged, the green fluorescence in HeLa and MCF-7 cancer cells gradually increased, suggesting that **3** and **5** could effectively induce ^1^O_2_ generation in cells. As shown in Figures 4E and 4F, the induced fluorescence intensity caused by **3** was 1.99 times higher than that caused by **5** for HeLa cells, and 1.71 times for MCF-7 cells under irradiation with green LED (40 mW/cm^2^) for 4 min. This meant that **3** containing heavy iodine atoms was more efficient in intracellular ^1^O_2_ production.

### *In Vitro* PDT Experiment

**Figure 5:**
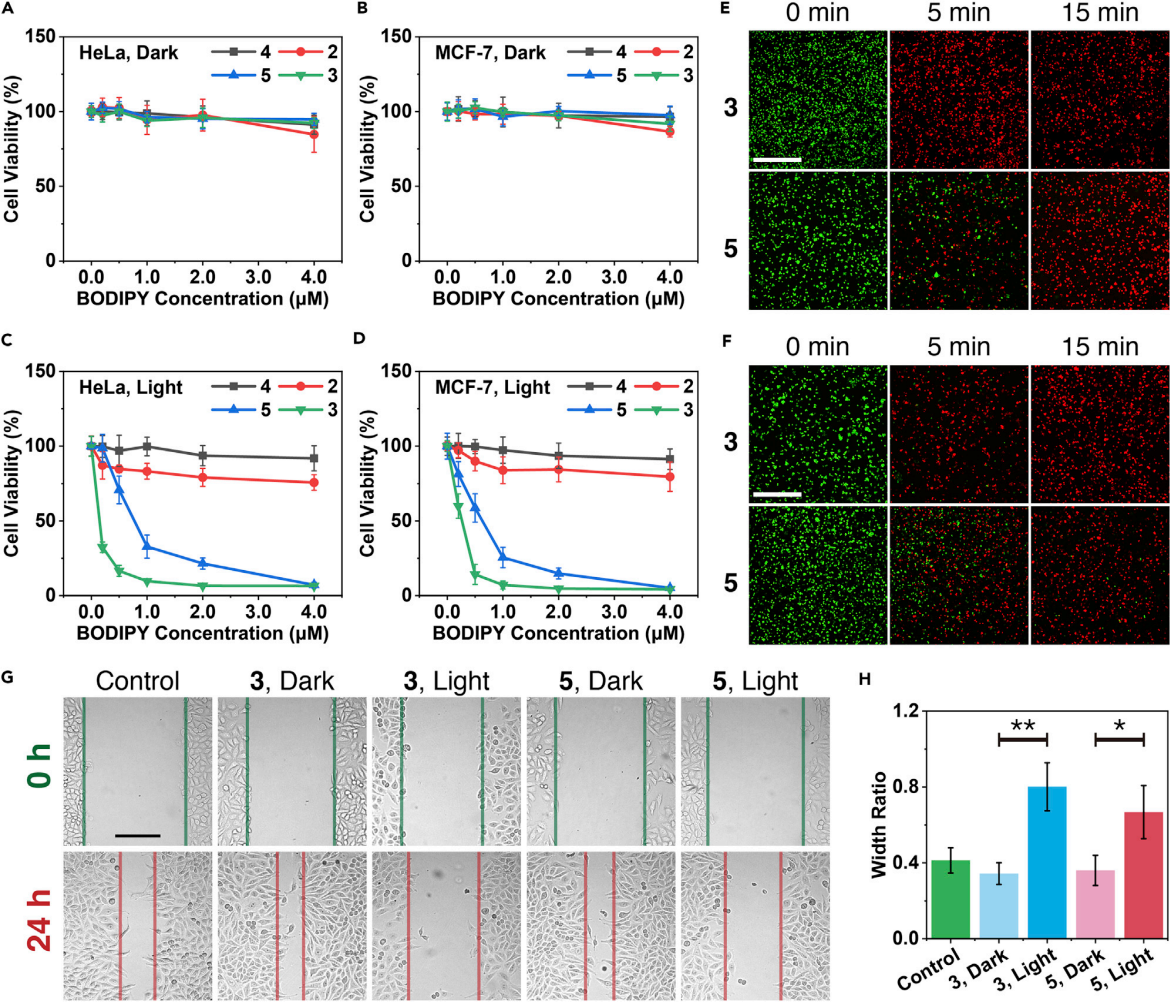
*In Vitro* PDT Induced by **3** and **5** (A–D) MTT assays of HeLa (A and C) and MCF-7 (B and D) cancer cells incubated with **2**, **3**, **4**, and **5**. Cells were incubated with DPBS dispersion of **2**, **3**, **4**, or **5** (100 μL, 0, 0.2, 0.5, 1.0, 2.0, 4.0 μM, BODIPY equiv.) for 30 min. Then the cells were exposed to green LED (40 mW/cm^2^) for 0 min (A and B) and 15 min (C and D), respectively. After additional 24-h incubation, the relative cell viabilities were detected by the standard MTT assay. Data were presented as mean ± SD (n = 5). (E and F) Laser scanning confocal images of HeLa (E) or MCF-7 (F) cells co-stained with calcein-AM (green, live cells) and propidium iodide (red, dead cells) after being incubated with **3** or **5** (2.0 μM, BODIPY equiv.) with green LED irradiation (40 mW/cm^2^) for 0, 5, 15 min, respectively. Scale bar, 500 μm. (G and H) *In vitro* scratch assays. MCF-7 cell monolayer with scratches was incubated with **3** or **5** (500 μL, 0.5 μM, BODIPY equiv.) for 30 min. Then cells were exposed to green LED (40 mW/cm^2^) for 0 or 5 min, respectively. The cells that were not incubated with **3** and **5** were used as controls. Representative images (G) and scratch width ratio of 0 h and 24 h (H) are shown. Scale bar, 200 μm. Data were presented as mean ± SD (n = 3, **p < 0.01, *p < 0.05).

The dark toxicities of **2**, **3**, **4**, and **5** were evaluated before intracellular PDT (see *In Vitro* PDT Experiment in Transparent Methods for experimental details). As shown in Figures 5A and 5B, the standard 3-(4,5-dimethyl-2-thiazolyl)-2,5-diphenyl-2*H*-tetrazolium bromide (MTT) assay (van Meerloo et al., 2011) showed that the cell viability for the HeLa and MCF-7 cancer cells induced by **2**, **3**, **4**, and **5** was more than 80%, even with a high BODIPY concentration up to 4.0 μM, indicating that their dark toxicity was negligible. Detailed study by the standard MTT assay indicated that **2**, **3**, **4,** and **5** exhibited very different phototoxicity for the cells under the given conditions (green LED, 40 mW/cm^2^, 15 min). As shown in Figures 5C and 5D, the free BODIPY molecule of **2** with heavy iodine atoms exhibited very weak phototoxicity to HeLa and MCF-7 cells. The cell viability of HeLa and MCF-7 was up to 75.7% ± 5.2% and 79.6% ± 9.8% even with 4.0 μM of **2** under the light irradiation. Meanwhile, the free BODIPY molecule of **4** without iodine atoms showed even lower phototoxicity under the same conditions. The corresponding cell viability of HeLa and MCF-7 was more than 90%. This poor phototoxicity of **2** and **4** was clearly attributed to their poor water solubility and cell membrane permeability. In contrast to **2** and **4**, **3** and **5** exhibited excellent phototoxicity. The HeLa cell viability was sharply down to 32.4% ± 3.5% even with concentration of **3** as low as 0.2 μM (BODIPY equiv.). For MCF-7 cells, a low cell viability of 14.3% ± 6.7% was observed with concentration of **3** at 0.5 μM (BODIPY equiv.). Compared with **3**, **5** without heavy atoms exhibited a lower phototoxicity. For example, the cell viability of HeLa and MCF-7 was 32.8% ± 7.7% and 25.6% ± 6.9% with 1.0 μM **5** (BODIPY equiv.). Thus the nanocrystallization of **2** and **4** via COF **1** significantly enhanced their cell membrane permeability and intracellular phototoxicity, and consequently, made their practical PDT application available.

The PDT efficacity of **3** and **5** was further confirmed by calcein-AM and propidium iodide (PI) double staining (Guo et al., 2017b) (see Calcein-AM/PI Double Stain in Transparent Methods for experimental details). As shown in Figures 5E and 5F, after incubation with **3** or **5** (2.0 μM BODIPY equiv.) for 30 min, the proportion of the dead HeLa and MCF-7 cells (red ones) significantly increased with the prolongation of irradiation time. This result again demonstrated that **3** and **5** possessed excellent PDT efficacy, but with ignorable dark toxicity.

As we know, invasiveness was one of the important features of cancer (Altorki et al., 2019, Stuelten et al., 2018). MCF-7 cells' migrating ability damage caused by PDT was assessed using cell scratch assays (see *In Vitro* Scratch Assay in Transparent Methods for experimental details), which could to some extent mimic the *in vivo* cell migration (Liang et al., 2007). As shown in Figures 5G and 5H, after incubation with **3** and **5** and subsequent light irradiation, only few MCF-7 cells migrated, whereas the invasive ability of the unilluminated MCF-7 cells was not affected by **3** and **5**. Therefore, we could conclude that the PDT induced by **3** and **5** can significantly decrease the MCF-7 cell migration *in vitro*.

### Cellular Uptake Mechanism

**Figure 6:**
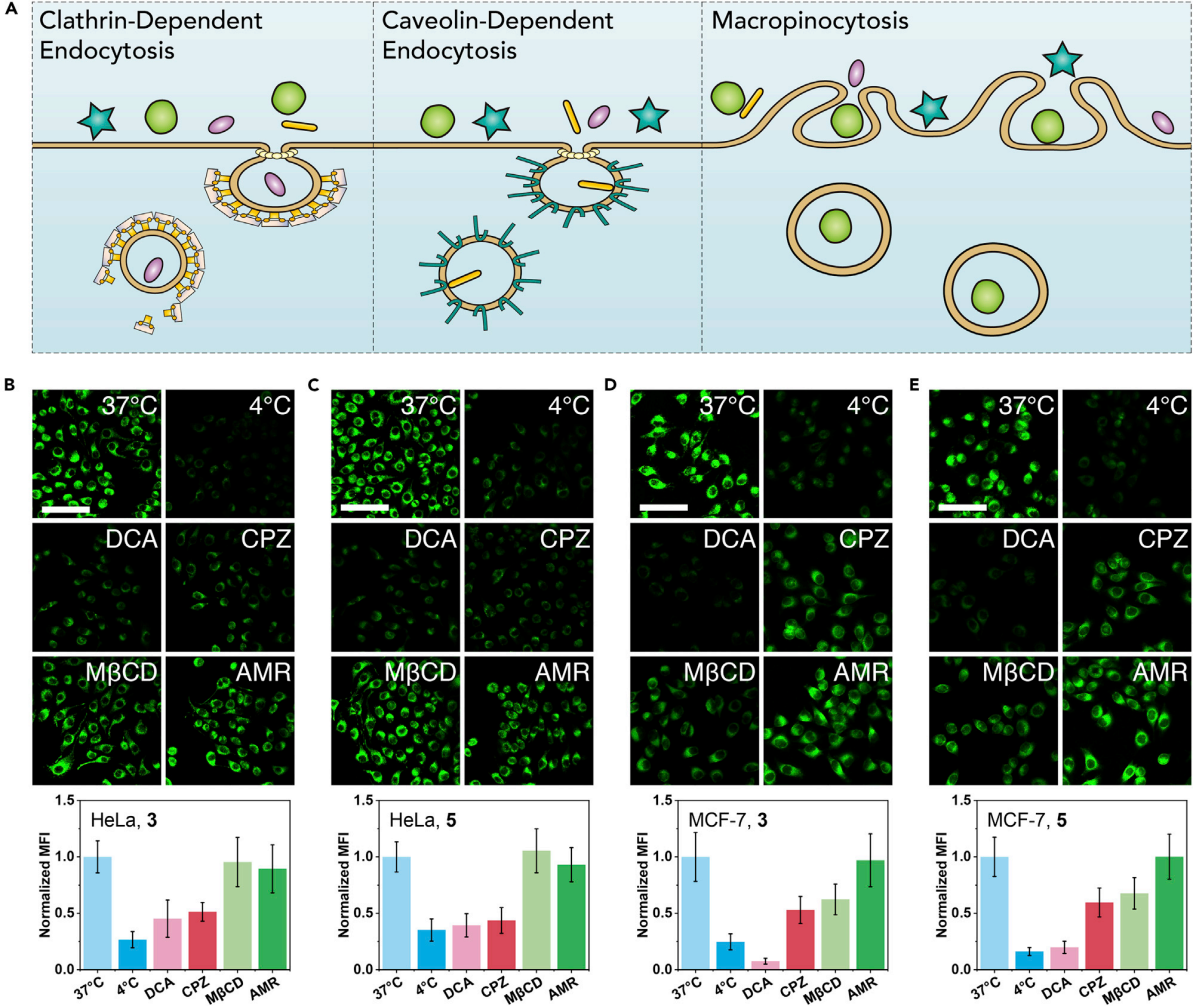
Cellular Uptake Mechanism of **3** and **5** (A) Energy-dependent pathways of cellular internalization of NPs. (B–E) Cellular uptake mechanism of **3** (B and D) and **5** (C and E) in HeLa (B and C) and MCF-7 (D and E) cells. The cells were treated with **3** or **5** (5 μg/mL) for 30 min, at 37°C, 4°C, and 37°C while pre-treating with DCA (15 mM), CPZ (10 μg/mL), MβCD (10 mg/mL), and AMR (75 μg/mL) for 1 h. The cellular uptake was reflected by MFI of green fluorescence. Data were presented as mean ± SD (n = 5). Scale bar, 100 μm.

The excellent PDT efficacity of **3** and **5** inspired us to explore the intrinsic mechanism of cell death (Figure 6, see Cellular Uptake Mechanism in Transparent Methods for experimental details). As a result, low temperature (4°C) and dichloroacetate (DCA, inhibiting aerobic glycolysis through inhibiting pyruvate dehydrogenase kinase; Kato et al., 2007, Nayak et al., 2018) inhibited cell uptake of **3** and **5**. This suggested that cellular uptake of **3** and **5** was an energy-dependent process and was closely related to the energy provided by aerobic glycolysis metabolism in cancer cells. Considering the energy-dependent pathway of cellular uptake by NPs (Mayor and Pagano, 2007, Shi and Tian, 2019), cells were pre-treated with chlorpromazine (CPZ, clathrin-dependent endocytosis inhibitor), amiloride (AMR, micropinocytosis inhibitor), and methyl-β-cyclodextrin (MβCD, caveolin-dependent endocytosis inhibitor), and then incubated with **3** or **5**. We found that HeLa and MCF-7 cells showed different cell uptake inhibition. For HeLa, CPZ significantly inhibited cellular uptake, demonstrating HeLa uptake of **3** and **5** by clathrin-dependent endocytosis. For MCF-7, CPZ and MβCD significantly inhibited cellular uptake, implying MCF-7 uptake of **3** and **5** by both clathrin-dependent endocytosis and caveolin-dependent endocytosis. AMR had no significant effect on the cell uptake, indicating that micropinocytosis has a very limited contribution to the cell uptake herein.

### Subcellular Localization

**Figure 7:**
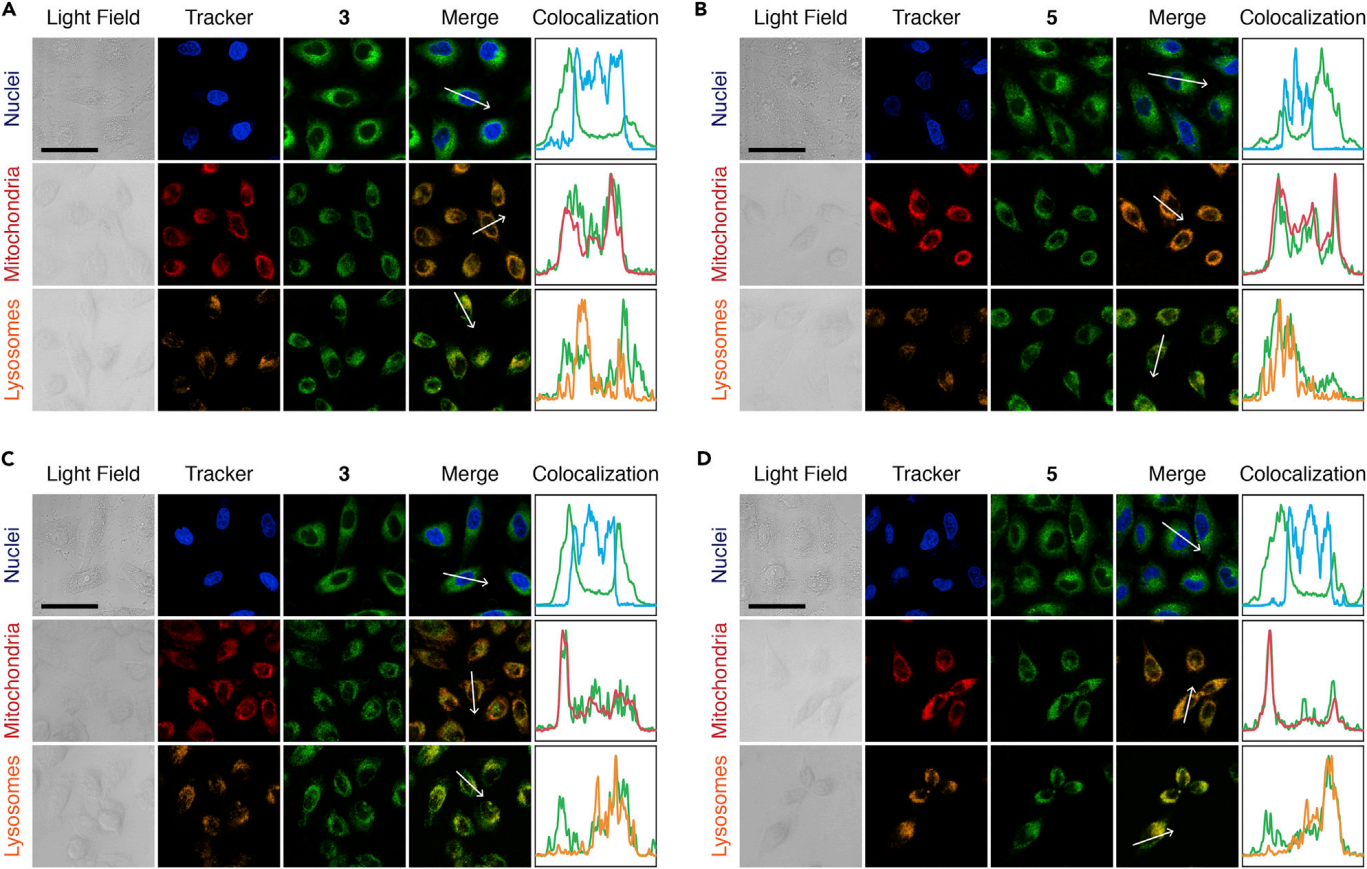
Subcellular Localization Studies of **3** and **5** (A–D) Subcellular localization of **3** (A and C) and **5** (B and D) in HeLa (A and B) and MCF-7 (C and D) cancer cells. HeLa and MCF-7 cancer cells were pretreated with **3** or **5** (5 μg/mL, 30 min), and subsequently co-incubated with Hoechst 33258 (5 μg/mL, 15 min), MitoTracker Deep Red FM (25 nM, 15 min), and LysoTracker red DND-99 (50 nM, 15 min). The fluorescence intensity profiles along the line segments in merged images showed that **3** and **5** colocalized with mitochondria and lysosomes, instead of nucleus. Scale bar, 50 μm.

Cell death caused by PDT was directly related to the ^1^O_2_-generated location (Gomes-da-Silva et al., 2018, Liu et al., 2011). Owing to the high local concentration of the *in situ*-formed ^1^O_2_, cellular structures with high PS concentrations were preferentially damaged by PDT, which induced subsequent cellular events. In view of this consideration, the distribution of **3** and **5** in cells was examined (Figure 7). After incubation with **3** and **5**, the nuclei, mitochondria, and lysosomes of the cancer cells were labeled with Hoechst 33258, MitoTracker Deep Red FM, and LysoTracker Red DND-99, respectively (see Subcellular Localization of Cell Nucleus, Subcellular Localization of Mitochondria, and Subcellular Localization of Lysosomes in Transparent Methods for experimental details). Laser confocal imaging showed that **3** and **5** were basically not localized in the nucleus, but mainly colocalized with lysosomes and mitochondria. We therefore assumed that **3** and **5** might induce cell death through the lysosome- (Kroemer and Jäättelä, 2005) and mitochondrion-associated pathways (Fulda et al., 2010) instead of the nucleus-associated pathway. Lysosomes are the main organelles that decompose exogenous NPs, and destruction of lysosomes can prevent NPs from being degraded (Dong et al., 2018, Gyparaki and Papavassiliou, 2014). Mitochondria are major contributors to endogenous reactive oxygen species and are closely related to oxidative stress and apoptosis in tumor cells (Vyas et al., 2016a, Wong et al., 2017). Therefore targeting mitochondria and lysosomes may be beneficial for efficient PDT.

### Cell Death Mechanism

**Figure 8:**
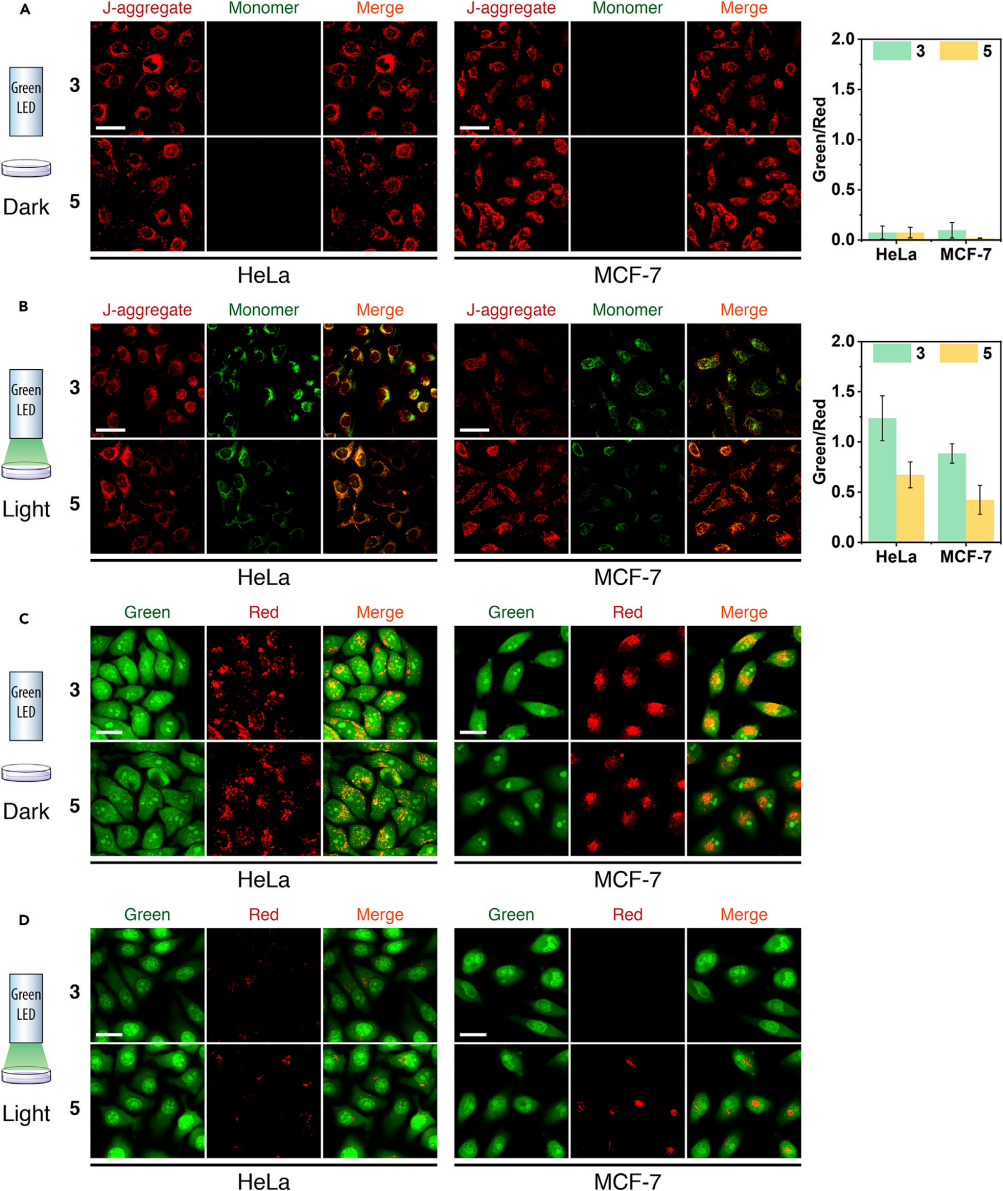
Characterization of Mitochondrial Membrane Potential (*ΔΨ*) Reduction and Lysosomal Membrane Permeabilization (LMP) Induced by **3** and **5** (A and B) Laser scanning confocal images of cells stained with JC-1 (10 μg/mL, 10 min) after being incubated with **3** or **5** (0.2 μM, BODIPY equiv.) with green LED irradiation (40 mW/cm^2^) for 0 min (A) and 4 min (B), respectively. *ΔΨ* reduction was reflected by the MFI ratios of green and red fluorescence. Data are presented as mean ± SD (n = 5). Scale bar, 50 μm. (C and D) Laser scanning confocal images of cells stained with AO (5 μg/mL, 10 min) after being incubated with **3** or **5** (0.2 μM, BODIPY equiv.) with green LED irradiation (40 mW/cm^2^) for 0 min (C) and 4 min (D), respectively. Scale bar, 25 μm.

Mitochondrial membrane potential (MMP, *ΔΨ*) was a hallmark event related to cell death by the mitochondrion-associated pathway (Burke, 2017, Weinberg and Chandel, 2015). The *ΔΨ* detection can be easily achieved by JC-1 (see Mitochondrial Membrane Potential (MMP, *ΔΨ*) in Transparent Methods for experimental details), which was a reliable fluorescent probe for *ΔΨ* (Lv et al., 2016, Tan et al., 2018). As shown in Figures 8A and 8B, after incubation with **3** and **5** (0.2 μM, BODIPY equiv.), only red fluorescence of JC-1 was observed without light irradiation owing to the formation of JC-1 *J*-aggregate, indicating that the *ΔΨ* was normal and **3** or **5** was not dark toxic. When irradiated with green LED, the JC-1 fluorescence changed from red to green due to the JC-1 monomer formation, demonstrating that a significant *ΔΨ* decrease occurred and the cell death caused by **3** and **5** was through the mitochondrion-associated pathway. It was worth noting that **3**-induced fluorescence intensity was significantly stronger than that of **5**, further indicating the heavy-atom effect on photosensitization.

In addition, the lysosome-associated-pathway-induced cell death was also examined based on lysosomal membrane permeabilization (LMP) (Marques et al., 2013). For this, acridine orange (AO) was selected as the fluorescent dye to perform the experiment (see Lysosomal Membrane Permeabilization (LMP) in Transparent Methods for experimental details). As shown in Figures 8C and 8D, a significant intracellular red dot fluorescence was observed due to the formation of protonated AO in the intact lysosomes (pH 4–5), implying that **3** and **5** did not damage the lysosomes in dark. However, the intracellular red fluorescence was weakened or even disappeared under light irradiation, which demonstrated that AO was released from the lysosome into the cytoplasm (pH ∼7.2), and moreover, deprotonated in the weak alkaline environment to emit diffuse green fluorescence (Al-Eisawi et al., 2016, Boya and Kroemer, 2008). This observation suggested that **3-** and **5**-triggered ^1^O_2_ can induce LMP, and effectively promoted cell death via lysosome-associated pathway. Again, **3** exhibited more effective LMP because of the heavy-atom effect.

### *In Vivo* PDT Experiment

**Figure 9:**
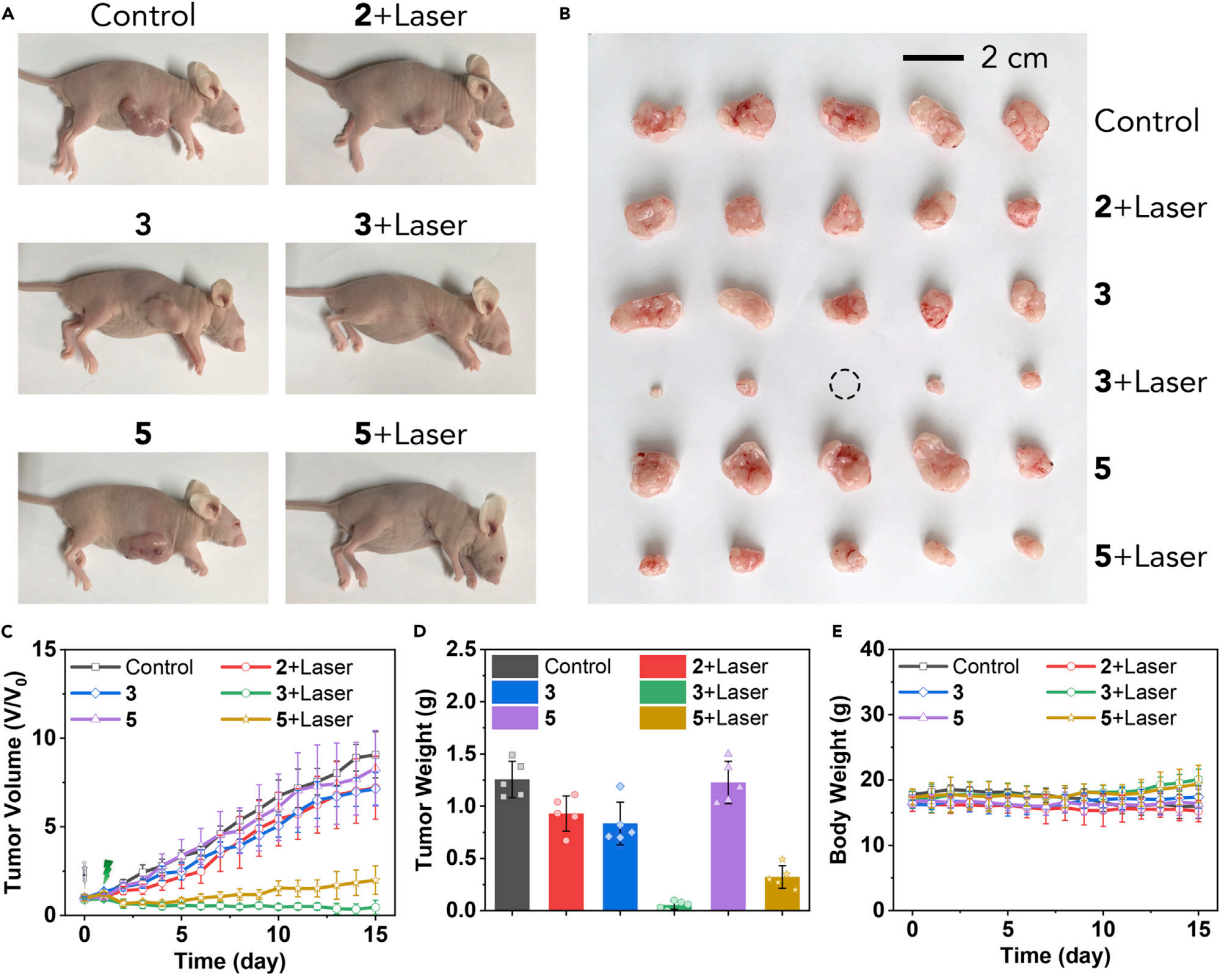
*In Vivo* PDT Experiment Animals were used in PDT experiments when the tumor size reached ∼150 mm^3^. Day 0, intratumoral injection; day 1, for the treatment group, light treatment was performed on the tumor site. The mice continued to be fed for 14 days. See MCF-7 Xenograft Model and *In Vivo* PDT Experiment in Transparent Methods for experimental details. (A) Representative photographs of MCF-7 tumor-bearing nude mice at the end of the treatment. (B) Photographs of tumor tissue obtained after treatment. Scale bar, 2 cm. (C) Tumor volume of the nude mice in various groups during the treatment. Data are presented as mean ± SD (n = 5). (D) Tumor weight obtained after treatment. Data are presented as mean ± SD (n = 5). (E) Body weight of the mice in various groups during the treatment. Data are presented as mean ± SD (n = 5).

All the aforementioned results proved that **3** and **5** were excellent COF-based nano-PSs. This encouraged us to continue to examine their *in vivo* anti-tumor ability (see MCF-7 Xenograft Model and *In Vivo* PDT Experiment in Transparent Methods for experimental details). As shown in Figures 9A–9D, tumors in nude mice treated with Dulbecco's phosphate-buffered saline (DPBS) increased rapidly. For the groups injected with **3** and **5** without light irradiation, a similar tumor growth trend was observed, but there was no necrosis of any tissue. This indicated that **3** and **5** were negligibly toxic to the biological tissues. For the group injected with **2** with light irradiation (green LED, 1.0 W/cm^2^), some slight signs of tumor inhibition effect were observed at the early treatment stages but then quickly recurred, and eventually there was no significant difference from the control group. The results of the **2** + laser group ruled out the individual role of the small molecular BODIPY and green laser irradiation in tumor inhibition. As expected, **3-** and **5**-induced PDT effectively inhibited tumor growth under green laser irradiation (1.0 W/cm^2^), and no recrudescent signs were observed during the whole experimental period. Compared with the **5** + laser group, the results from **3** + laser group strongly suggested that the heavy-atom effect can effectively enhance the *in vivo* PDT efficacy, which is entirely consistent with their *in vitro* PDT examination.

Besides, Figure 9E showed that all nude mice with intratumor injection presented a steady growth or no change in their body weight, whereas the body weight of the control group was slightly decreased. Histopathological examination revealed that there was no substantial damage or distinct inflammation lesions in organs, including the heart, liver, spleen, lung, and kidney (Figure S10, see Histopathological Examination in Transparent Methods for experimental details). All these results confirmed that **3** and **5** had negligible adverse impact and favorable biocompatibility on organisms and were indeed suitable for *in vivo* anti-tumor application.

## Discussion

COFs have picked up intense attention of researchers worldwide due to their promising applications in heterogeneous catalysis (Ding and Wang, 2013, Tu et al., 2018, Yan et al., 2018), energy storage (Cao et al., 2019, Xiao and Xu, 2018, Zhang et al., 2017b, Zheng et al., 2019, Zhou and Wang, 2017), analytical chemistry (Qian et al., 2018b, Zhang et al., 2018d, Zhang et al., 2019), and light conversion (Banerjee et al., 2018). As an emerging class of organic crystalline materials, COFs offered some specific advantages not found in other types of materials: modularity, porosity, stability, and metal free (Chandra et al., 2013, Kandambeth et al., 2012). The application of COFs in biomedical field, especially cancer therapeutics, however, is still in its infancy owing to the difficulty in their size- and structure-controlled synthesis and poor aqueous dispersibility (Li and Yang, 2017) (see Table S1 for details). On the other hand, the bulky and active organic PSs, such as BODIPY herein, were sometimes hard to be safely anchored onto the COF framework by the bottom-up approach especially under the rigorous solvothermal conditions. In addition, the covalent decoration of PSs on the established COF framework by the existing PSM approach (Ma and Scott, 2018) might be limited by the stability and activity of the pre-embedded active precursors under the COF synthetic conditions (Xu et al., 2016), and also the incoming bulky functional moiety of PSs might lead to crystallinity decrease, structural transformation, and even structure collapse of the COF pristine, although some very impressive PSM examples have been reported very recently (Bunck and Dichtel, 2013, Chen et al., 2014, Daugherty et al., 2019, Dong et al., 2016, Guo et al., 2017a, Haase et al., 2018, Han et al., 2018, Huang et al., 2015a, Huang et al., 2015b, Li et al., 2018c, Li et al., 2019, Lohse et al., 2016, Lu et al., 2018, Mitra et al., 2017, Nagai et al., 2011, Qian et al., 2017, Qian et al., 2018a, Rager et al., 2017, Sun et al., 2017, Sun et al., 2018a, Sun et al., 2018b, Vardhan et al., 2019, Waller et al., 2016, Waller et al., 2018, Xu et al., 2014, Xu et al., 2015a, Xu et al., 2015b) (see Table S2 for details).

**Figure 10:**
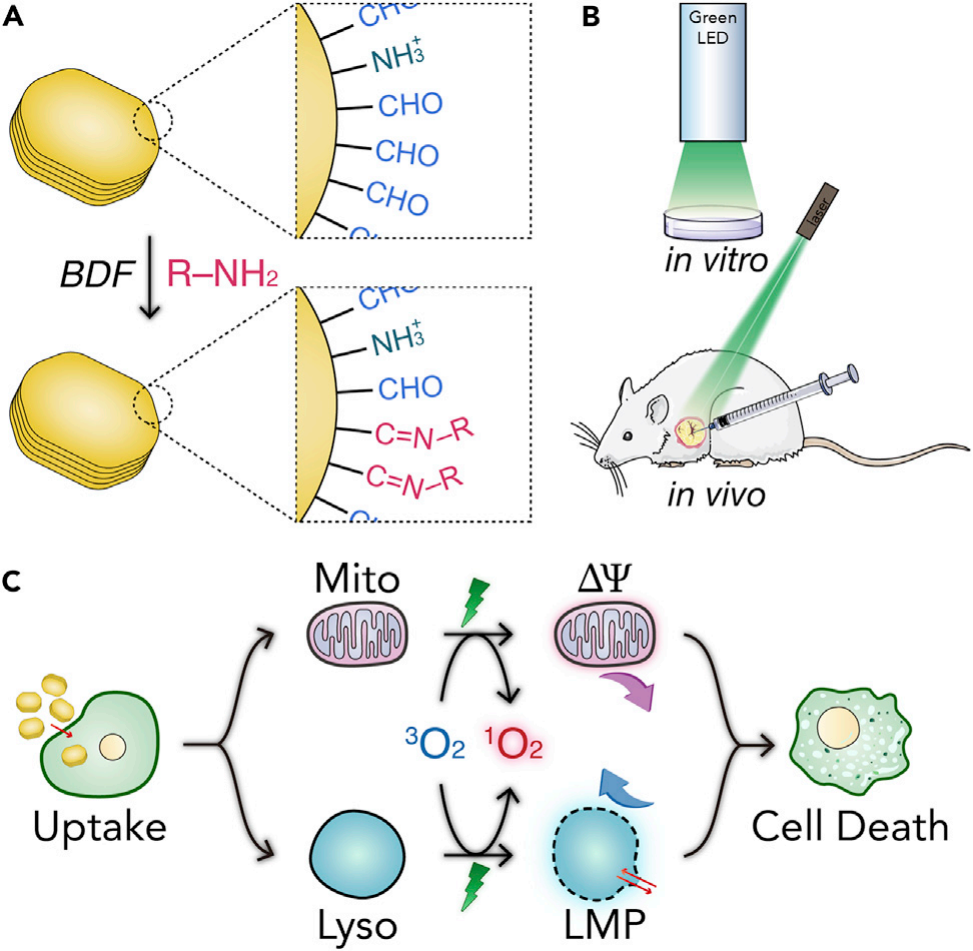
Bonding Defects Functionalization Strategy of Nanoscale COFs for PDT (A) Diagram of bonding defect functionalization (BDF) of NCOF LZU-1 (**1**). (B) Diagram of *in vitro* and *in vivo* PDT. (C) Diagram of **3-** and **5-**induced cell death mechanism.

On the other hand, COFs could be considered as a type of porous crystalline organic “reactive end-group polymers.” In principle, these intrinsic bonding defects in COF matrix can be further modified and tailored to the desired application via BDF approach. As shown in Figure 10A, by reaction of the free end –CHO group on **1** with –NH_2_ moiety on **2** or **4**, the organic PSs of BODIPY were covalently attached to the COF NPs via Schiff-base condensation under the given conditions to generate the NCOF-based PSs. As a consequence, the organic BODIPY components have been successfully nanocrystallized without altering the pristine COF structure and the inherent property of BODIPY; moreover, the possible BODIPY leaching was also effectively avoided due to the covalent decoration. Indeed, both **3** and **5** featured a clear chemical composition and excellent chemical, aqueous, and photostability. In addition, their nanometer size (ca. 110 nm), low dark toxicity, and extremely high phototoxicity made them become attractive NCOF-based PSs for PDT, which was well evidenced by the *in vitro* and *in vivo* experiments (Figure 10B).

In *in vitro* experiments, we took the HeLa and MCF-7 cancer cell lines to examine the PDT of **3** and **5** in detail. Intracellular ^1^O_2_ imaging, cytostatic, and calcein-AM/PI double staining tests all supported that **3** and **5** are excellent nanomedical agents for PDT, especially **3** with heavy atoms of iodine. Furthermore, the spread of cancer cells was effectively inhibited by **3-** and **5-**involved PDT, as revealed by *in vitro* scratch assays. Notably, the *in vitro* PDT performance exhibited by **3** was better than those of reported BODIPY-based PDT systems (see Table S3 for detailed comparative analysis). Their high-efficacy PDT, especially **3**, was further confirmed by the *in vivo* experiments, and they are able to effectively inhibit the MCF-7 xenograft growth without detectable systemic toxicity. More detailed mechanism study (Figure 10C) showed that **3** and **5** were taken up by cancer cells via energy-dependent endocytosis, and that they were mainly localized to lysosomes and mitochondria, and consequently led to cell death through mitochondrion- and lysosome-associated pathways.

### Conclusion

We can reasonably assume that the BDF approach herein is flexible and versatile, and that it can be used to construct new types of NCOF-based nanomedicines by covalently grafting various functional species such as bioactive, chemotherapeutic, and targeting agents, and so on via bonding defects decoration. In doing so, the functionality of the COF-based materials could be significantly enriched, and their application scope would be effectively expanded. To the best of our knowledge, this is the first example of NCOF-based materials for PDT. In the past two decades, nanotherapeutics, as an emerging medical treatment approach, has provided a promising way for clinical cancer treatment (Chen et al., 2017, Hartshorn et al., 2018, Wang et al., 2018b). A wide variety of nanomaterials (Croissant et al., 2018, Yang et al., 2018, Zhang et al., 2018a) have been developed for cancer therapeutics. PDT as a minimally invasive method has received much more attention due to its unique tissue selectivity (Agostinis et al., 2011, Li et al., 2018b). We believe that the NCOF-based PDT materials via BDF herein with both nano- and organic PS merits not only expanded the type of nanomaterials for PDT but also might open up new avenues for the design and fabrication of many more new nanomedicines, especially for cancer therapy.

### Limitations of Study

This study focused on the free end –CHO on LZU-1 by BDF, and no chemical modification on the free –NH_2_ of LZU-1 NP was performed. If so, other types of functional molecules such as antineoplastic drugs might be further grafted on the NCOF by BDF. In doing so, more advanced and effective NCOF-based medicines would be achieved.

## Methods

All methods can be found in the accompanying Transparent Methods supplemental file.

## Supplemental Information

Document S1. Transparent Methods, Figures S1–S11, and Tables S1–S3
