# Supplementary material for: BODIPY-Decorated Nanoscale Covalent Organic Frameworks for Photodynamic Therapy
Source: iScience. 2019 Mar 29;14:180–98. doi: 10.1016/j.isci.2019.03.028 (PMC6461589; doi:10.1016/j.isci.2019.03.028)
Supplement: Document S1. Transparent Methods, Figures S1–S11, and Tables S1–S3 [file mmc1.pdf]

ISCI, Volume *14*

Supplemental Information

BODIPY-Decorated Nanoscale Covalent Organic Frameworks for Photodynamic Therapy

Qun Guan, Dan-Dan Fu, Yan-An Li, Xiang-Mei Kong, Zhi-Yuan Wei, Wen-Yan Li, Shao-Jun Zhang, and Yu-Bin Dong

Supplemental Figures

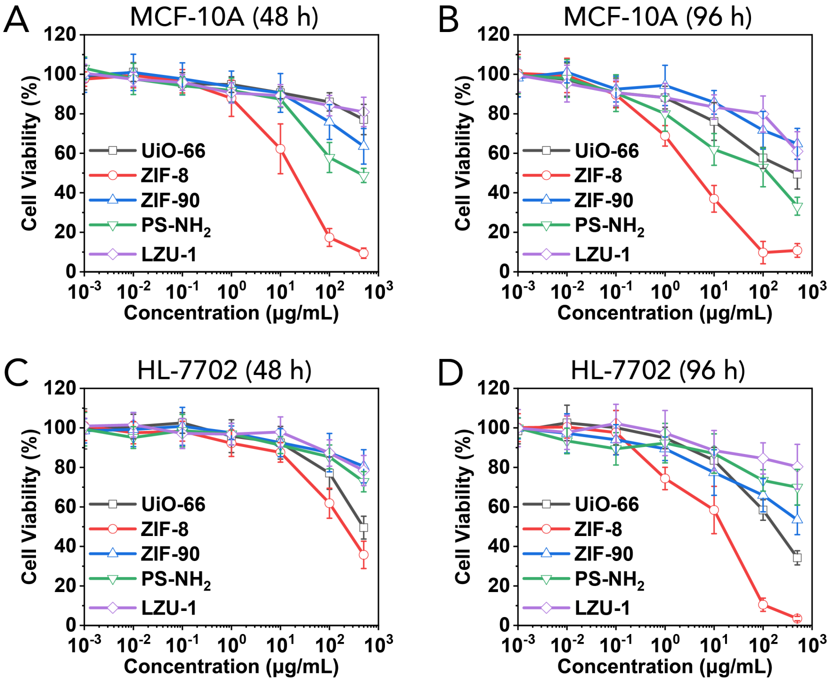

Figure S1. Cytotoxicity of Common Polymeric Nanoparticles to Normal Tissue Cell Lines, Related to Figure 1.

Human mammary epithelial cell MCF-10A (A, B) and human normal liver cell HL-7702 (C, D) were cultured with the medium supplemented with LZU-1 (**1**), UiO-66, ZIF-8, ZIF-90, and PS-NH_2 for 48 h (A, C) or 96 h (B, D). Then, the relative cell viabilities were detected by the standard MTT assay. Data was presented as mean±SD (n=4).

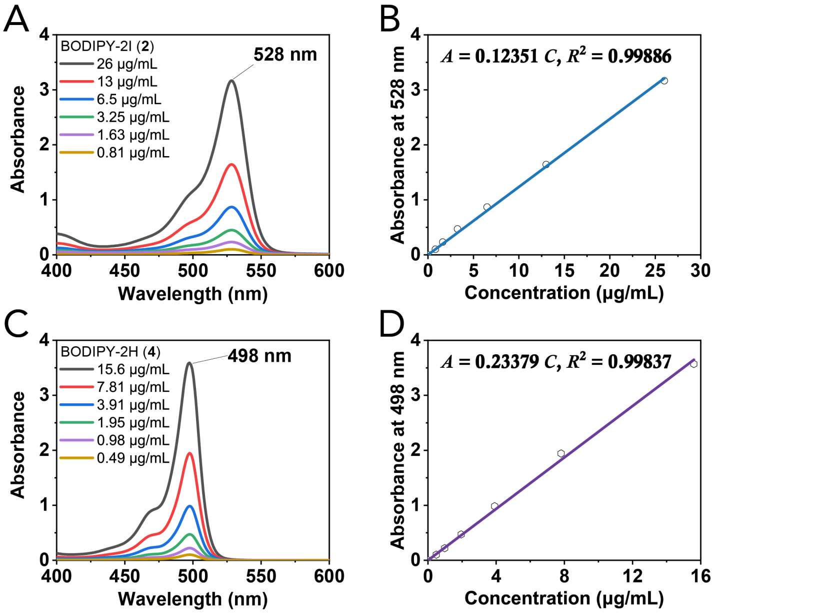

Figure S2. Standard Curves of **2** and **4**, Related to Figure 1.

(A, C) UV-vis spectra of **2** (A) and **4** (C).

(B, D) Standard curves of **2** (B) and **4** (D).

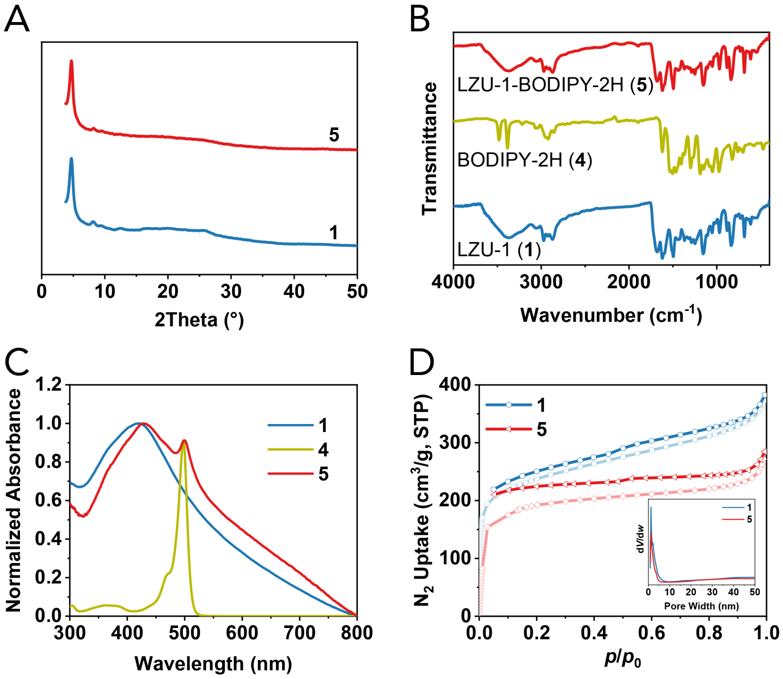

Figure S3. Spectroscopy Characterization of **1** and **5**, Related to Figure 2.

(A) Powder X-ray diffraction (PXRD) patterns of **1** and **5**.

(B) FT-IR spectra of **1**, **4**, and **5** powders.

(C) UV-vis absorption spectra of **1**, **4**, and **5** in DMF.

(D) N_2 adsorption-desorption isotherms (77 K) of **1** and **5**. Inset images: pore width distribution plots.

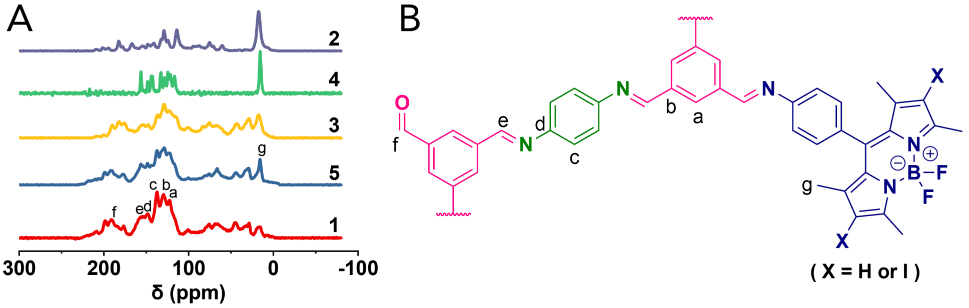

Figure S4. ^{13}C ss-NMR spectra of **1**, **2**, **3**, **4**, and **5**, Related to Figure 2.

(A) ^{13}C ss-NMR spectra of **1**, **2**, **3**, **4**, and **5**.

(B) Ownership of major ^{13}C ss-NMR peaks.

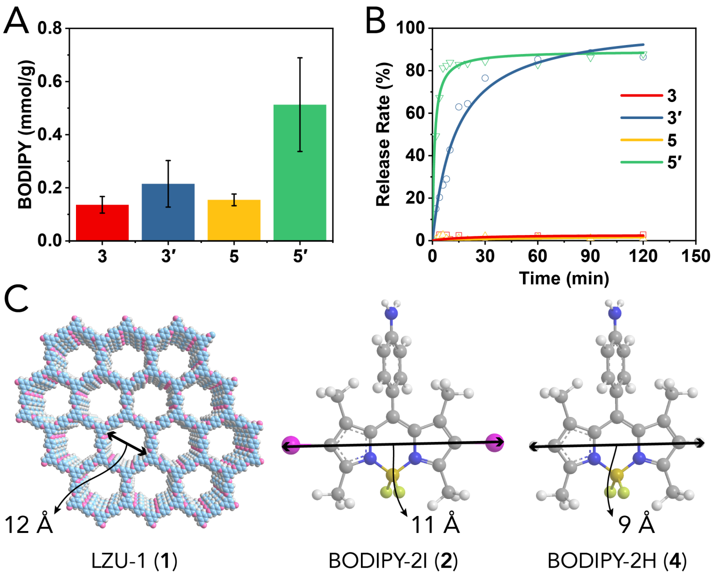

Figure S5. Control Experiments, Related to Figure 2.

(A) The content of BODIPY in different samples. Data was presented as mean±SD (n=3).

(B) BODIPY release rate as a function of time.

(C) Pore width of **1** and molecular dimensions of **2** and **4**.

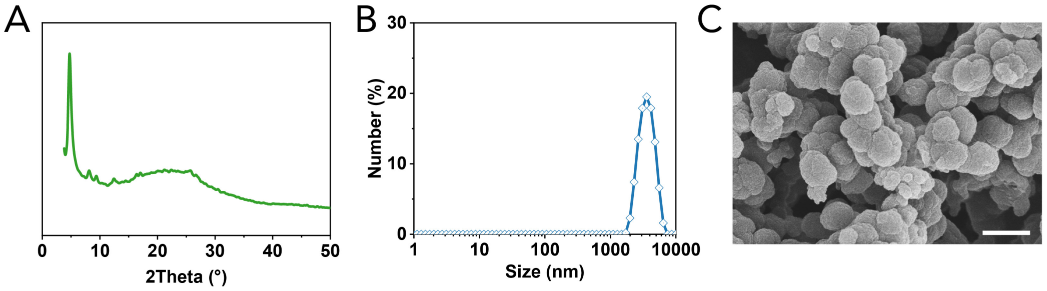

Figure S6. Characterization of the LZU-1 which was generated from the direct reaction of TFB and *p*-phenylenediamine under the same solvothermal conditions, Related to Figure 3.

(A) PXRD pattern of micron sized LZU-1.

(B) DLS size profiles of micron sized LZU-1 in PBS (pH=6.5).

(C) SEM images of micron sized LZU-1. Scale bar: 1 μm.

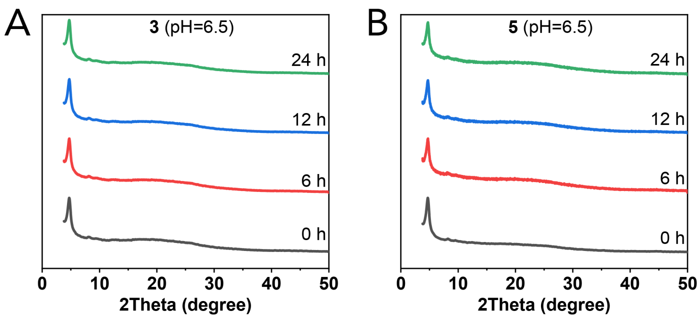

Figure S7. Chemical Stability of **3** and **5**, Related to Figure 2.

(A) PXRD of **3** after soaking in PBS (pH=6.5) for different time.

(B) PXRD of **5** after soaking in PBS (pH=6.5) for different time.

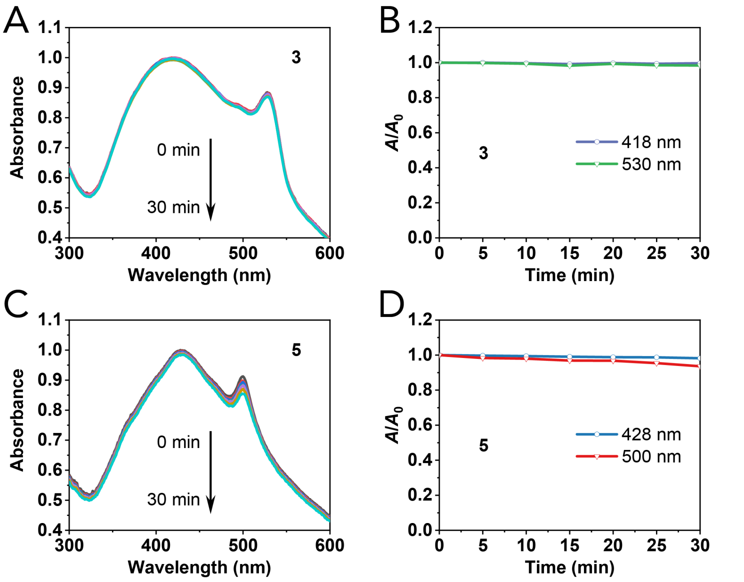

Figure S8. Light Stability of **3** and **5**, Related to Figure 2.

(A) UV−vis spectra of **3** after exposure to green laser for different time.

(B) Absorbance at 418 nm and 530 nm of **3** as functions of illumination time.

(C) UV−vis spectra of **5** after exposure to green laser for different time.

(D) Absorbance at 428 nm and 500 nm of **5** as functions of illumination time.

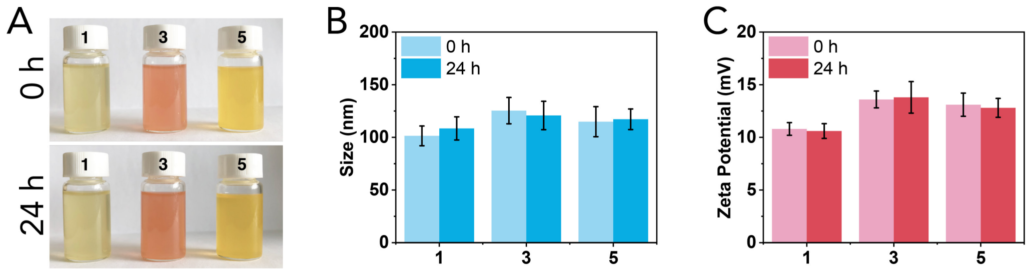

Figure S9. Colloidal Stability of **1**, **3**, and **5** PBS Dispersion, Related to Figure 2.

(A) Digital photos of **1**, **3**, and **5** PBS (pH=6.5) dispersion before and after standing for 24 h.

(B) Particle sizes measured by DLS of **1**, **3**, and **5** PBS (pH=6.5) dispersion before and after standing for 24 h. Data was presented as mean±SD (n=3).

(C) Zeta potentials of **1**, **3**, and **5** PBS (pH=6.5) dispersion before and after standing for 24 h. Data was presented as mean±SD (n=3).

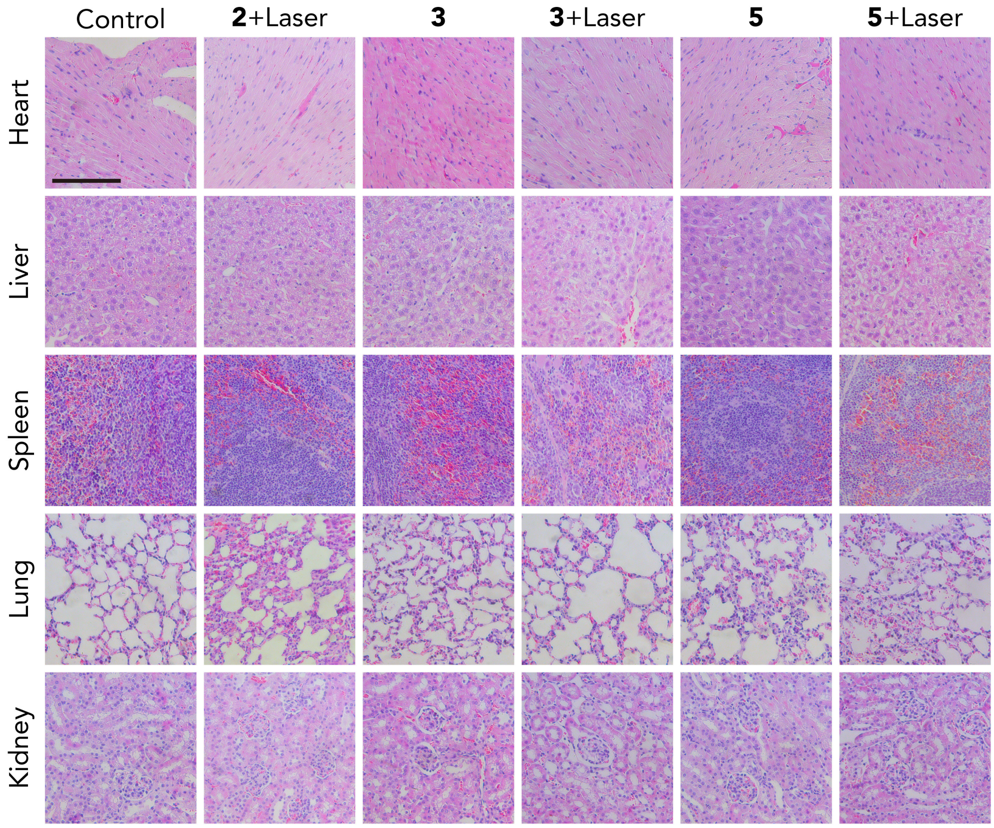

Figure S10. H&E Stained Images, Related to Figure 9.

H&E stained images of the major organs, including heart, liver, spleen, lung, and kidney. Scale bar: 100 μm.

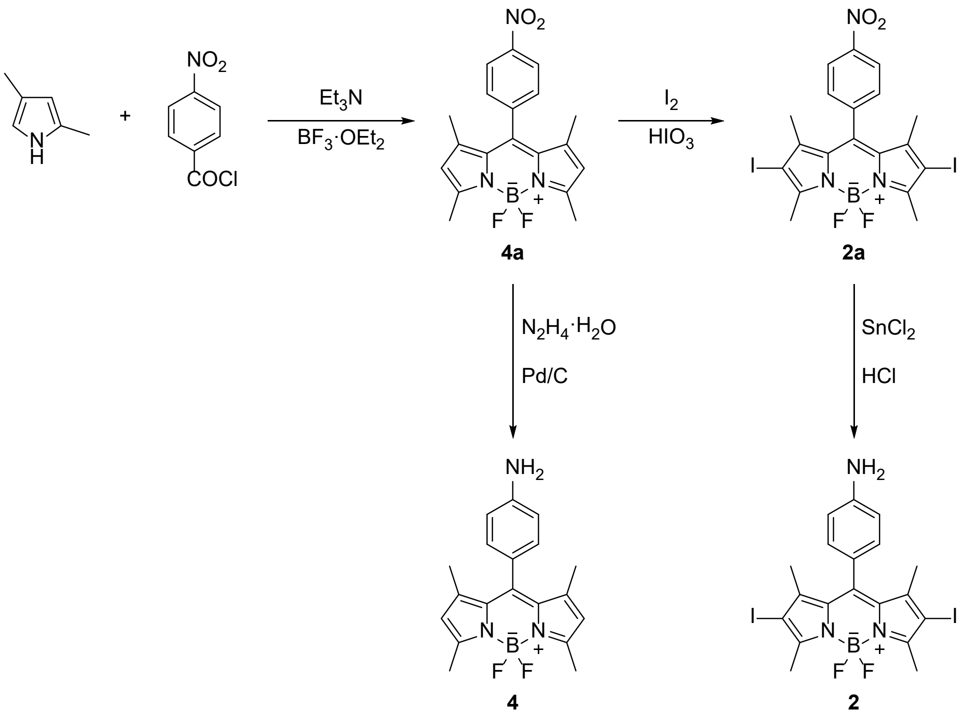

Figure S11. Synthetic Routes of **2** and **4**, Related to Figure 1.

Supplemental Tables

Table S1. Summary of Recent Typical COF-Based Biomedicine Systems, Related to Figure 10.

COF materials	Particle size	Application	Results	References
TpASH-FA-5FU	1~1.5 μm, nanosheets	Drug delivery	Three-step post-synthetic modification<br>Lack of *in vivo* experiments	(Mitra et al., 2017)
PEG-CCM@APTES-COF-1	~200 nm	Drug delivery	Complex surface modification based on PEG<br>Limited water-dispersion and anti-tumor effects	(Zhang et al., 2018)
TTI-COF	Several micrometers	Drug delivery	Poor dispersion<br>Limited anti-tumor effects	(Vyas et al., 2016)
PI-2-COF & PI-3-COF	<1 μm	Drug delivery	Serious aggregated<br>Need to add DMSO to assist dispersion<br>Lack of *in vivo* experiments	(Bai et al., 2016)
PI-COF-4 & PI-COF-5	Several micrometers	Drug delivery	Just a concept<br>Serious aggregated<br>Lack of *in vitro* & *in vivo* experiments	(Fang et al., 2015)
DOX@COF	100~150 nm	Drug delivery	Poor stability in PBS	(Liu et al., 2019a)
TpASH-NPHS	~90 nm, nanosheets	Fluorescence bioimaging	Complex solvent-assisted liquid sonication process<br>Multi-step post-synthetic modification	(Wang et al., 2018)
TTA-DFP	~22 nm, nanosheets	Fluorescence bioimaging	Complex solvent-assisted liquid sonication process	(Das et al., 2018)
COF-TpMA	~1 μm	Fluorescence sensing	Serious aggregated<br>Need to add DMSO to assist dispersion<br>Slight cytotoxicity<br>Lengthy manual grinding	(Liu et al., 2019b)
TpTta	Several micrometers	Fluorescence sensing	Just a concept<br>Serious aggregated<br>Lack of *in vitro* & *in vivo* experiments	(Li et al., 2017)
Fe_3O_4@COF	~500 nm	Photothermal conversion	Just a concept<br>Template method<br>Lack of *in vitro* & *in vivo* experiments	(Tan et al., 2016)
EDTFP-1	~200 nm, nanofibres	Chemotherapy	Toxicity of COF itself<br>Serious aggregated	(Bhanja et al., 2017)
$TpTG_{Cl}$	~200 nm, nanosheets	Antibacterial	Toxicity of COF itself<br>Complex solvent-assisted liquid sonication process	(Mitra et al., 2016)
3D-TPP	Coatings, unknown thickness	$^1O_2$ generation	COF coatings instead of dispersions<br>Illumination time >24 h<br>Photodynamic antibacterial	(Hynek et al., 2018)
COFs-Trif-Benz	Several micrometers	$^1O_2$ generation	Poor dispersion; illumination time >1 h<br>Photodynamic antibacterial	(Liu et al., 2017a)
3D-Por-COF	~1 μm	$^1O_2$ generation	Just a concept<br>Illumination time >1 h<br>Lack of *in vitro* & *in vivo* experiments	(Lin et al., 2017)
ZnTPP-CuPc-COF	Several micrometers	$^1O_2$ generation	Just a concept<br>Serious aggregated<br>Lack of *in vitro* & *in vivo* experiments	(Feng et al., 2016)

COF materials	Particle size	Application	Results	References
CuP-SQ	~1 μm	$^{1}O_2$ generation	Just a concept<br>Serious aggregated<br>Lack of *in vitro* & *in vivo* experiments	(Nagai et al., 2013)
LZU-1-BODIPY-2H (**5**)	~110 nm	Real PDT		**This work**
LZU-1-BODIPY-2I (**3**)	~110 nm	Real PDT		**This work**

Table S2. Summary of COFs Post-Synthetic Modification Methods, Related to Figure 10.

Monomer Modification	References
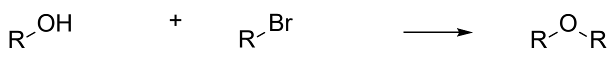	(Dong et al., 2016)
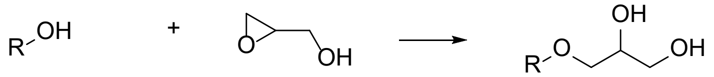	(Mitra et al., 2017)
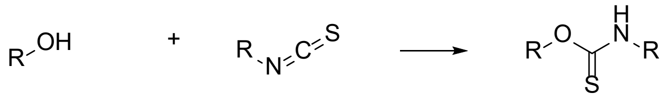	(Rager et al., 2017)
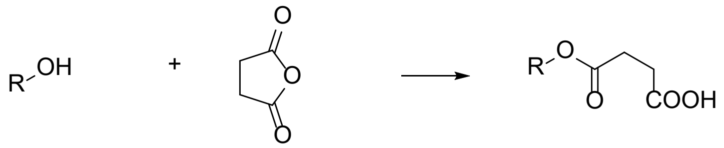	(Huang et al., 2015a; Lu et al., 2018)
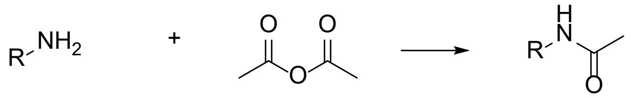	(Lohse et al., 2016)
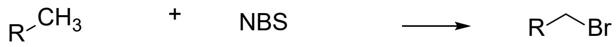	(Guo et al., 2017)
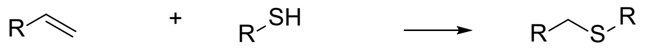	(Bunck and Dichtel, 2013; Sun et al., 2017; Sun et al., 2018b)
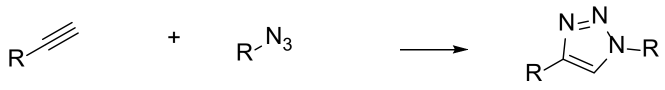	(Chen et al., 2014; Huang et al., 2015b; Nagai et al., 2011; Xu et al., 2015a; Xu et al., 2014; Xu et al., 2015b)
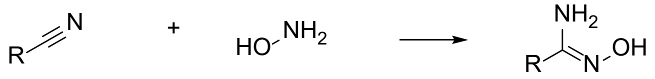	(Sun et al., 2018a)
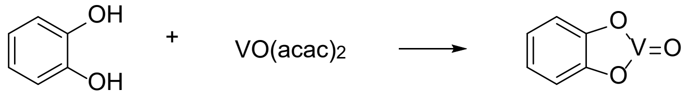	(Vardhan et al., 2019)
**Monomer Exchange**	
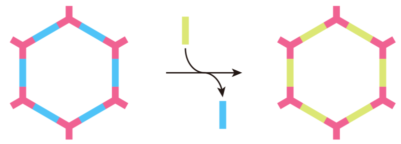	(Qian et al., 2017; Qian et al., 2018)
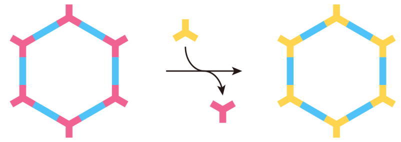	(Daugherty et al., 2019)
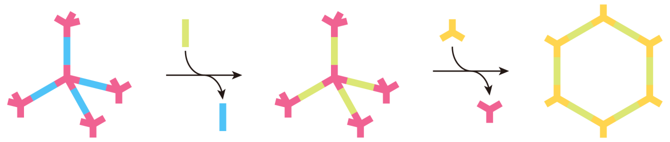	(Li et al., 2019)

Imine Bond Modification	
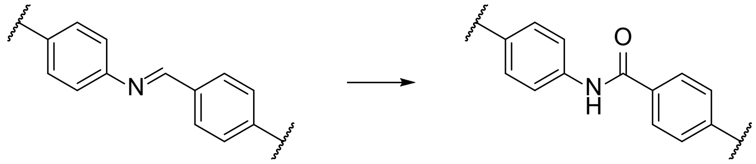	(Han et al., 2018; Waller et al., 2016)
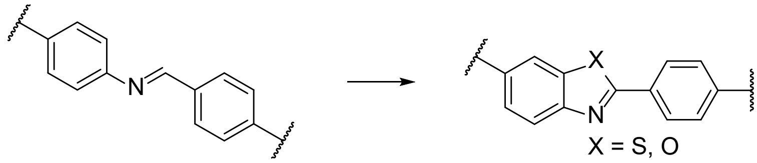	(Haase et al., 2018; Waller et al., 2018)
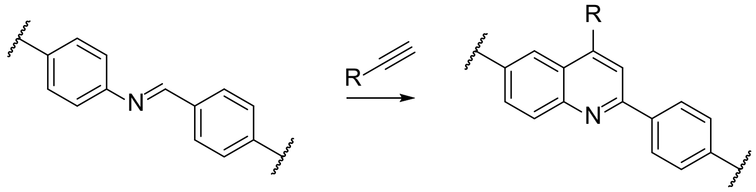	(Li et al., 2018)
**Bonding Defects Functionalization**	
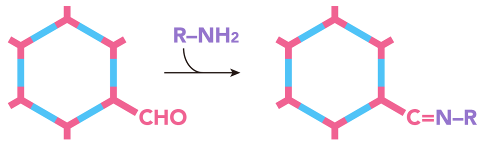	This work

Table S3. Summary of Recent Typical BODIPY-Based PDT Systems in Cancer Cells, Related to Figure 10.

Sample	Types	Light Source	Cells	T (min)	C (μM)	CV	References
pH-PDT	small molecules	532 nm laser	HepG2	4	2.0	~30%	(Xue et al., 2018)
BODIPY	small molecules	620 nm laser	HeLa	40	2.5	~20%	(Liu et al., 2017b)
RET-BDP	small molecules	600~800 nm laser	HeLa	60	13.2	~50%	(Huang et al., 2017)
Bodiplatin-NPs	small molecules	660 nm laser	4T1	5	6.0	~20%	(Guo et al., 2016)
Car-BDP-TNM	small molecules	Halogen lamp	HeLa	30	9.1	~20%	(Huang et al., 2016)
polymer/BODIPY-Br2	Polymer NPs	635 nm lamp	HepG2	10	5.4	~40%	(Liu et al., 2016)
BODIPY@ZIF-90	MOF NPs	Green LED	HepG2	8	6.1	~20%	(Guan et al., 2018)
UiO-PDT	MOF NPs	Visible light	B16F10	10	0.7	~25%	(Wang et al., 2016)
LZU-1-BODIPY-2H (**5**)	COF NPs	Green LED	HeLa	15	2.0	~21%	This work
LZU-1-BODIPY-2H (**5**)	COF NPs	Green LED	MCF-7	15	2.0	~15%	This work
LZU-1-BODIPY-2I (**3**)	COF NPs	Green LED	HeLa	15	0.5	~16%	This work
LZU-1-BODIPY-2I (**3**)	COF NPs	Green LED	MCF-7	15	0.5	~14%	This work

* T: lighting time; C: PS concentration; CV: cell viability; NPs: nanoparticles.

Transparent Methods

Materials, Instrumentations, and Cell Culture

All reactants were reagent grade and were used as purchased without further purification. 2,4-Dimethyl-1*H*-pyrrole, benzene-1,3,5-tricarbaldehyde (TFB), trifluoroacetic acid (TFA), boron trifluoride ethyl ether complex ($BF_3 \cdot Et_2O$), iodine, iodic acid, stannous chloride (anhydrous), polyvinyl pyrrolidone (PVP, Mw=8000), 2-methyl-1*H*-imidazole (MeIm), 1*H*-imidazole-2-carbaldehyde (IcaH), $Zn(NO_3)_2 \cdot 6H_2O$, trioctylamine (TOA), $ZrCl_4$, terephthalic acid, and amine-modified polystyrene microsphere ($PS\text{-}NH_2$, 0.05~0.1 μm) were purchased from Aladdin Reagent Co., Ltd. Palladium on activated charcoal (Pd, 10 wt%), hydrazine hydrate ($N_2H_4 \cdot H_2O$, 85 wt%), sodium sulfate (anhydrous), triethylamine (TEA), and benzene-1,4-diamine (PDA) were purchased from Sinopharm Chemical Reagent Co., Ltd. *tert*-Butyl (4-aminophenyl)carbamate (NBPDA) was purchased from Ark Pharm, Inc. 4-Nitrobenzoyl chloride and 1,3-diphenylisobenzofuran (DPBF) were purchased from TCI (Shanghai) Development Co., Ltd. All organic solvents were purchased from Sinopharm Chemical Reagent Co., Ltd. Dehydrated solvents were obtained after treating solvents with standard procedures. Ultra-pure water was prepared with an Aquapro System (18 MΩ).

Chlorpromazine hydrochloride (CPZ), methyl-β-cyclodextrin (MβCD), and amiloride hydrochloride (AMR) were purchased from MedChemExpress Co. Ltd. Sodium dichloroacetate (DCA) and 3-(4,5-dimethyl-2-thiazolyl)-2,5-diphenyl-2*H*-tetrazolium bromide (MTT) were purchased from Sigma-Aldrich (Shanghai) Trading Co. Ltd. Phosphate-Buffered Saline (PBS), Dulbecco's Phosphate-Buffered Saline (DPBS), and Hank's Balanced Salt Solution (HBSS) were purchased from Biological Industries USA, Inc. Dulbecco's Modified Eagle Medium (DMEM), Fetal Bovine Serum (FBS), Penicillin Streptomycin Mixtures (Pen-Strep), and Trypsin-EDTA Solution (0.25%) were purchased from HyClone Laboratories, Inc. Normocin was purchased from Invivogen (San Diego, CA, USA). Mammary Epithelial Cell Growth Basal Medium (MEBM) and Mammary Epithelial Cell Growth Medium (MEGM) SingleQuots Kit were purchased from Lonza Inc.

Singlet Oxygen Sensor Green (SOSG), LysoTracker Red DND-99, MitoTracker Deep Red FM, Hoechst 33258, and JC-1 were purchased from Thermo Fisher Scientific Inc. Calcein-AM/PI Double Stain Kit was purchased from Yeasen Biotech (Shanghai) Co., Ltd. Acridine orange (AO) and formalin fixative were purchased from Beijing Solarbio Science & Tecnology Co., Ltd.

Liquid-state ^{1}H and ^{13}C nuclear magnetic resonance (NMR) spectra were recorded using a Bruker AVANCE III HD 400 MHz NMR Spectrometer. Chemical shifts were reported as δ values relative to tetramethylsilane (TMS) as internal reference. Solid-state ^{13}C NMR spectra (cross-polarization magic angle spinning) were recorded using an Agilent VNMRS 600 MHz NMR Spectrometer. Chemical shifts were reported as δ values relative to TMS as internal reference. MALDI-TOF mass spectra were recorded using a Bruker BIFLEX III Ultra-High-Resolution Fourier Transform Ion Cyclotron Resonance (FT-ICR) Mass Spectrometer. Ultraviolet–visible absorption spectra were recorded on a Shimadzu UV-2700 Double Beam UV-Vis Spectrophotometer. Fourier transform infrared (FT-IR) spectra were obtained in the 4000~400 cm^{-1} range using a Thermo Scientific Nicolet iS50 FT-IR Spectrometer equipped with single reflection diamond ATR module. Elemental microanalyses (EA) were performed with an Elementar Vario EL Cube Elemental Analyzer. Scanning electron microscopy (SEM) micrographs were recorded on a Hitachi SU8010 Scanning Electron Microscope. Transmission electron microscope (TEM) micrographs were recorded on a Hitachi HT7700 120kV Compact-Digital Transmission Electron Microscope. Powder X-ray

diffraction (PXRD) patterns were obtained on a Bruker D8 ADVANCE X-Ray Powder Diffractometer with Cu Kα line focused radiation (λ=1.5405 Å) from 2ϑ=3.8° up to 50.0° with 0.01° increment. Nitrogen isotherms were measured at 77 K using a Micromeritics ASAP2020 HD88 Surface Area and Porosity Analyser. Before measurement, the samples were degassed in vacuum at 120°C for 12 h. Hydrodynamic particle size and zeta potential were measured using Malvern Zetasizer Nano ZS90 System. Laser scanning confocal fluorescence images were captured with a Leica TCS SP8 Confocal Laser Scanning Microscopy with an objective lens (×20, ×10). Microplate assays were carried out on a Molecular Devices SpectraMax i3x Multi-Mode Microplate Detection System.

The HeLa (human cervical cancer cell line), MCF-7 (human breast adenocarcinoma cell line), and HL-7702 (human normal liver cell line) were provided by Institute of Basic Medicine, Shandong Academy of Medical Sciences (Jinan, China). The MCF-10A (human mammary epithelial cell line) was provided by Stem Cell Bank, Chinese Academy of Sciences (Shanghai, China). The HeLa, MCF-7, and HL-7702 cell lines were cultured in DMEM supplemented with FBS (10%), Normocin (50 μg/mL), penicillin (100 U/mL) and streptomycin (100 μg/mL) in an atmosphere of 5% CO_2 and 95% air at 37°C. The MCF-10A cell line was cultured in MEBM supplemented with MEGM SingleQuots Kit in an atmosphere of 5% CO_2 and 95% air at 37°C.

Synthesis of NCOF LZU-1 (**1**)

A mixture of benzene-1,3,5-tricarbaldehyde (20 mg, 123 μmol), *tert*-butyl (4-aminophenyl)carbamate (40 mg, 192 μmol), PVP (160 mg, Mw=8000), and trifluoroacetic acid (720 μL) in ethanol (8 mL) was heated at 120°C for 12 h under autogenous pressure. Then, the reaction system was cooled to room temperature. After 5 days standing, the particles were isolated by centrifugation at 12000 rpm for 30 min. The solid was washed with ethanol/triethylamine (v/v=9:1) for three times, and washed with ether for additional one time. Finally, the solids were dried in air at 40°C to generate **1** nanoparticle as claybank powder. Yield: ~10 mg. FT-IR (cm^{-1}): 3358 (m), 2971 (m), 2871 (m), 1682 (m), 1621 (s), 1495 (s), 1440 (m), 1368 (m), 1288 (m), 1249 (m), 1155 (s), 1052 (w), 970 (w), 883 (w), 837 (m), 732 (w), 685 (m), 615 (w).

Normal Tissue Cytotoxicity Test

For demonstrating the biocompatible of COF, we evaluated the growth inhibition of the obtained nano LZU-1 herein on normal tissue cell lines (human normal liver cell line HL-7702, and human mammary epithelial cell line MCF-10A), furthermore, compared with other widely used polymeric nanocarriers, e.g. UiO-66, ZIF-8, ZIF-90 and amine-modified polystyrene microsphere (PS-NH_2). As shown in Figure S1, when using medium supplemented with different NPs for continuous cell culture, for the MCF-10A cell line, ZIF-8 showed the greatest cytotoxicity at 48 h, while LZU-1, UiO-66, and ZIF-90 exhibited the similar toxicity, the cell viabilities were still >80% at concentrations up to 100 μg/mL. When the culture time was extended to 96 h, the cytotoxicity of LZU-1 and ZIF-90 was still at a low level, while the toxicity of UiO-66 and PS-NH_2 was slightly increased. For the HL-7702 cell line, ZIF-8 and UiO-66 had higher cytotoxicity at 48 h, while the toxicity of LZU-1 and ZIF-90 was almost negligible, even at concentrations up to 500 μg/mL. When the culture time was extended to 96 h, LZU-1 showed the lowest cytotoxicity among the UiO-66, ZIF-8, ZIF-90, and PS-NH_2. These results are consistent with the previous reports.(Guan et al., 2018; Jiang et al., 2019; Ruyra et al., 2015; Tamames-Tabar et al., 2014) This suggested that the metal-free inherent nature of COFs such as LZU-1 herein provided an excellent biocompatibility and minimal normal tissue toxicity, which might be one of the key advantages of COFs for biomedical applications.

Experimentally, HL-7702 and MCF-10A cells were cultured with the medium supplemented with LZU-1 (**1**), PS-NH_2, ZIF-8, ZIF-90, and UiO-66 (100 μL, 0~500 μg/mL) at 48-well plates in CO_2 incubator for 48 h or 96 h. Then, the relative cell viabilities were detected by the standard MTT assay.(van Meerloo et al., 2011) ZIF-8(Zheng et al., 2016), ZIF-90(Guan et al., 2018), and UiO-66(Zhou et al., 2018) were synthesized according to previous report.

Synthesis of BODIPY-2I (**2**)

As shown in Figure S11, firstly, the precursor compound 5,5-difluoro-2,8-diiodo-1,3,7,9-tetramethyl-10-(4-nitrophenyl)-5*H*-4λ^4,5λ^4-dipyrrolo[1,2-*c*:2',1'-*f*][1,3,2]diazaborinine (**2a**) was synthesized as previously reported.(Guan et al., 2018) Next, under N_2 protection, **2a** (0.5 g, 0.8 mmol), anhydrous $SnCl_2$ (2.3 g, 12.0 mmol), hydrochloric acid (0.2 M, 12 mL) were dissolved in methanol (60 mL) and dichloromethane (60 mL). The mixture was refluxed for 12 h and then cooled to room temperature. Then, the mixture was washed with NaOH solution (1 M) and water. The precipitate and the aqueous layer were discarded, and the organic layer was dried over anhydrous sodium sulfate. The product was purified by alkaline alumina column chromatography (eluant, dichloromethane) to provide the product as deep red powder. Yield: 0.2 g (42%). ^{1}H NMR (400 MHz, $CDCl_3$) δ 6.97 (d, J = 8.3 Hz, 2H), 6.79 (d, J = 8.3 Hz, 2H), 3.90 (bs, 2H), 2.63 (s, 6H), 1.51 (s, 6H). ^{13}C NMR (101 MHz, $CDCl_3$) δ 156.26, 147.59, 145.42, 142.49, 131.95, 128.89, 124.22, 115.59, 85.35, 17.28, 15.99. MALDI-TOF MS, Calcd. For [M], 590.965, Found, 590.999; Calcd. For [M-F], 571.967, Found, 572.010. FT-IR (cm^{-1}): 3459 (m), 3378 (m), 3233 (w), 2924 (m), 2853 (w), 1625 (m), 1521 (s), 1455 (s), 1399 (s), 1343 (s), 1305 (s), 1264 (m), 1171 (s), 1117 (m), 1081 (m), 991 (s), 826 (m), 763 (m), 703 (m), 587 (m), 523 (m). Anal. Calcd. For $C_{19}H_{18}BF_2I_2N_3$ (%): C, 38.61; H, 3.07; N, 7.11, Found: C, 38.90; H, 3.17; N, 7.32.

Synthesis of LZU-1-BODIPY-2I (**3**)

1 (5 mg) and **2** (10.6 mg, 18 μmol) was added in ethanol (5 mL). The mixture was dispersed with ultrasonic dispersion for 10 min. Then acetic acid solution (50 μL, 3 M) was added, and the mixture was heated at 75°C for 4 h under autogenous pressure. Then, the reaction system was cooled to room temperature, and the particles were isolated by centrifugation at 12000 rpm for 30 min. The solid was washed with ethanol until the supernatant liquid was colorless, and washed with ether for additional one time. Finally, the solids were dried in air at 40°C to generate **3** as orange-red powder. FT-IR (cm^{-1}): 3368 (m), 2794 (m), 2926 (m), 2872 (m), 1696 (m), 1623 (s), 1599 (s), 1508 (s), 1455 (m), 1420 (w), 1393 (w), 1379 (m), 1344 (w), 1305 (w), 1287 (w), 1266 (w), 1251 (w), 1195 (w), 1158 (m), 1087 (w), 1052 (w), 972 (m), 885 (m), 835 (m), 764 (w), 733 (w), 687 (m), 617 (w), 587 (w), 521 (w).

Synthesis of BODIPY-2H (**4**)

As shown in Figure S11, firstly, the precursor compound 5,5-difluoro-1,3,7,9-tetramethyl-10-(4-nitrophenyl)-5*H*-4λ^4,5λ^4-dipyrrolo[1,2-*c*:2',1'-*f*][1,3,2]diazaborinine (**4a**) was synthesized as previously reported.(Guan et al., 2018) Next, under N_2 protection, **4a** (0.6 g, 1.63 mmol), hydrazine hydrate (15 mL, 85 wt%), Pd/C (2.0 g, 10 wt%) were dissolved in tetrahydrofuran (100 mL) and ethanol (100 mL). The mixture was refluxed for 12 h and then cooled to room temperature. The product was purified by alkaline alumina column chromatography (eluant, dichloromethane) to provide the product as red powder. Yield: 0.43 g (78%). ^{1}H NMR (400 MHz, $CDCl_3$) δ 7.00 (d, J = 8.4 Hz, 2H), 6.77 (d, J = 8.4 Hz, 2H), 5.97 (s, 2H), 3.88 (bs, 2H), 2.54 (s, 6H), 1.49 (s, 6H). ^{13}C NMR (101 MHz, $CDCl_3$) δ 154.90, 146.99, 143.20, 142.65, 131.99, 128.88, 124.60, 120.92, 115.42, 14.66, 14.55. MALDI-TOF MS, Calcd. For [M], 339.172, Found, 339.658; Calcd. For [M-F], 320.173, Found, 320.585. FT-IR (cm^{-1}): 3481 (m), 3384 (m), 3217 (w), 3066 (w), 2029 (w), 2952 (w), 2922 (w), 2856 (w), 1622 (m), 1537 (s), 1504 (s), 1468 (s), 1406 (s), 1369 (m), 1298 (s), 1265 (m), 1190 (s), 1151 (s), 1084 (s), 1051 (s), 976 (s), 820 (m), 762 (m), 731 (m), 702

(m), 580 (m), 472 (m). Anal. Calcd. For $C_{19}H_{20}BF_2N_3$ (%): C, 67.28; H, 5.94; N, 12.39, Found: C, 67.43; H, 6.11; N, 12.65.

Synthesis of LZU-1-BODIPY (5)

1 (5 mg) and **4** (6 mg, 18 μmol) was added in ethanol (5 mL). The mixture was dispersed with ultrasonic dispersion for 10 min. Then acetic acid solution (50 μL, 3 M) was added, and the mixture was heated at 75°C for 4 h under autogenous pressure. Then, the reaction system was cooled to room temperature, and the particles were isolated by centrifugation at 12000 rpm for 30 min. The solid was washed with ethanol until the supernatant liquid was colorless, and washed with ether for additional one time. Finally, the solids were dried in air at 40°C to generate **5** as orange powder. FT-IR (cm^{-1}): 3378 (m), 2970 (m), 2870 (m), 1682 (m), 1621 (s), 1495 (s), 1439 (m), 1368 (m), 1288 (m), 1250 (m), 1154 (s), 1050 (w), 970 (m), 882 (w), 838 (m), 732 (w), 686 (m), 614 (w).

BODIPY Contents Determination

After the end of the synthesis, the supernatants were collected by centrifugation, and all the washing liquids produced during the washing were collected. The contents of BODIPY in the supernatant and the washing liquid were calculated using standard curves. These contents were subtracted from the total reactants to obtain the BODIPY contents in **3** and **5**.

BODIPY contents in **3** and **5** were further confirmed by the ICP-OES results. **3** or **5** (25 mg) was dissolved in the mixed acid (5.0 mL, H_2SO_4/HNO_3, v/v=1:1). Subsequently, the solution was diluted with water to 50.0 mL, and the content of B was determined using ICP-OES.

Control Experiments

A mixture of **1** (5 mg) and **2** (10.6 mg, 18 μmol) in ethanol (5 mL) was dispersed with ultrasonic dispersion for 10 min. Then, water (50 μL) was added, and the mixture was stirred at room temperature for 4 h. Then, the particles were isolated by centrifugation at 12000 rpm for 30 min, and the obtained solids were dried in air to generate BODIPY-2I⊂LZU-1 (**3'**) nanoparticles as orange-red powder.

A mixture of **1** (5 mg) and **4** (6 mg, 18 μmol) in ethanol (5 mL) was dispersed with ultrasonic dispersion for 10 min. Then, water (50 μL) was added, and the mixture was stirred at room temperature for 4 h. Then, the particles were isolated by centrifugation at 12000 rpm for 30 min, and the obtained solids were dried in air to generate BODIPY-2H⊂LZU-1 (**5'**) nanoparticles as orange powder.

The content of BODIPY in different samples were shown in Figure S5A determined by the standard curve method (Figure S2). The release curves of BODIPY were determined as follows: **3**, **3'**, **5**, or **5'** (3 mg) were added to boiling ethanol (100 mL); dispersions were taken at different times to test UV-vis spectra and converted to the release rate of BODIPY using standard curves (Figure S2).

Chemical Stability

3 or **5** (10 mg) in PBS (10 mL, pH= 6.5) was centrifuged at different time, and dried to examine their powder X-ray diffraction (PXRD). For more information about chemical stability, see (Chandra et al., 2013; Kandambeth et al., 2012).

Light Stability

A PBS (pH=6.5) dispersion of **3** or **5** (2 mL, 50 μg/mL) was exposed to green laser (1 W/cm^2) at room temperature for 30 min. The UV−vis spectra were recorded at 5 min intervals. The ratios A/A_0 of absorbance A and the initial absorbance A_0 at 418, 530 nm for **3** or 428, 500 nm for **5** at different irradiation times were calculated and plotted as the ordinate for the irradiation time. PBS (pH=6.5) was used as the reference for this UV−vis measurement.

Colloidal Stability

A PBS (pH=6.5) dispersion of **1**, **3**, and **5** (50 μg/mL) were allowed to stand at room temperature for 24 h, and their zeta potentials and hydrodynamic particle sizes were measured by dynamic light scattering (DLS).

Singlet Oxygen Generation in PBS

Pipetted the PBS (pH=6.5) dispersions of **3** or **5** (2 mL, 10 μM, BODIPY equiv) into quartz cuvette, and DPBF DMF solution (100 μL, 1mM) was added. Then the mixture was exposed to green LED (40 mW/cm^2) at room temperature for 60 s. The absorbance of DPBF at 414 nm in the mixture was recorded at 10 s intervals. The $^1O_2$ generation rate was determined from the reduced the absorbance over time. To characterize the difference in the rate of $^1O_2$ produced by different samples, the ratios A/A_0 of absorbance A and the initial absorbance A_0 at 414 nm at different irradiation times were calculated and plotted as the ordinate for the irradiation time. PBS (pH=6.5) dispersions of **3** or **5** (2 mL, 10 μM, BODIPY equiv) was used as the reference for this UV−vis measurement.

Intracellular Singlet Oxygen Generation

Cells were incubated with DPBS dispersion of **3** or **5** (200 μL, 0.2 μM, BODIPY equiv) in CO_2 incubator for 30 min, washed with DPBS twice, and further incubated with SOSG (5 μM, 200 μL) for 15 min. The cells were exposed to green LED (40 mW/cm^2) for different times and imaged with a laser scanning confocal microscope. The green images were excited by 488 nm light, and the emission wavelength range was collected at 525±20 nm. The mean fluorescence intensity (MFI) was analyzed by ImageJ software.(Schneider et al., 2012)

In Vitro PDT Experiment

Cells were incubated with DPBS dispersion of **2**, **3**, **4** or **5** (100 μL, 0, 0.2, 0.5, 1.0, 2.0, 4.0 μM, BODIPY equiv) in CO_2 incubator for 30 min, and washed with DPBS twice. Then, the cells were exposed to green LED (40 mW/cm^2) for 0 or 15 min. After additional 24 h incubation, the relative cell viabilities were detected by the standard MTT assay.(van Meerloo et al., 2011)

Calcein-AM/PI Double Stain

Cells were incubated with DPBS dispersion of **3** or **5** (200 μL, 2.0 μM, BODIPY equiv) in CO_2 incubator for 30 min, and washed with DPBS twice. Then, the cells were exposed to green LED (40 mW/cm^2) for 0, 5, 15 min. After additional 4 h incubation, the cells were collected using Trypsin-EDTA Solution (0.25%), washed with DPBS twice carefully, and were stained with Calcein-AM (500 μL, 2 μM) and PI (500 μL, 4.5 μM) for 15 min. Finally, the cells were washed with DPBS twice carefully, and imaged with a laser scanning confocal microscope. The green images of living cells were excited by 488 nm light, and the emission wavelength range was collected at 520±20 nm. The red images of dead cells were excited by 488 nm light, and the emission wavelength range was collected at 640±20 nm.

In Vitro Scratch Assay

MCF-7 cells were seeded into 12-well plates and grown to confluence. Then, cell monolayer was damaged by scratching with a sterile 1000 μL pipet tip to obtain scratches. Cells were incubated with DPBS dispersion of **3** or **5** (500 μL, 0.5 μM, BODIPY equiv) in CO_2 incubator for 30 min, and carefully washed with DPBS twice. The 0 h reference images of the scratched areas were taken using inverted microscope. Then, the cells were exposed to green LED (40 mW/cm^2) for 0 or 5 min. After additional 24 h incubation, the scratched areas were taken again. The cells that were not incubated with **3** and **5** were used as a control. The scratch widths were measured by ImageJ software,(Schneider et al., 2012) and the width ratios of 0 h and 24 h were calculated. The data was the result of 3 independent experiments.

Cellular Uptake Mechanism

Cells were subjected to different treatments before the incubation of **3** and **5** as follow: (i) DPBS, CO_2 incubator, 1 h; (ii) HBSS, air atmosphere, 4°C, 1 h; (iii) sodium dichloroacetate (DCA), 15 mM, CO_2 incubator, 1 h; (iv) chlorpromazine (CPZ), 10 μg/mL, CO_2 incubator, 1 h; (v) methyl-β-cyclodextrin (MβCD), 10 mg/mL, CO_2 incubator, 1 h; (vi) amiloride (AMR), 75 μg/mL, CO_2 incubator, 1 h. After these different treatments, the cells were incubated with DPBS dispersion of **3** or **5** (200 μL, 5 μg/mL) in CO_2 incubator for 30 min, and washed with DPBS twice. After additional 4 h incubation, the laser scanning confocal fluorescence images were captured. The green images of **3** or **5** were excited by 488 nm light, and the emission wavelength range was collected at 540±20 nm. The mean fluorescence intensity (MFI) was analyzed by ImageJ software.(Schneider et al., 2012)

Subcellular Localization of Cell Nucleus

Cells were incubated with DPBS dispersion of **3** or **5** (200 μL, 5 μg/mL) in CO_2 incubator for 30 min, and washed with DPBS twice. After additional 4 h incubation, cells were fixed in paraformaldehyde fix solution (4%) for 15 min, washed with DPBS twice, subsequently incubated with Hoechst 33258 (200 μL, 5 μg/mL) for an additional 15 min, and washed with DPBS twice. Then, the laser scanning confocal fluorescence images were captured. The green images of **3** or **5** were excited by 488 nm light, and the emission wavelength range was collected at 540±20 nm. The blue images of cell nucleus were excited by 405 nm light, and the emission wavelength range was collected at 461±30 nm. Colocalization was analyzed by ImageJ software.(Schneider et al., 2012)

Subcellular Localization of Mitochondria

Cells were incubated with DPBS dispersion of **3** or **5** (200 μL, 5 μg/mL) in CO_2 incubator for 30 min, and washed with DPBS twice. After additional 4 h incubation, cells were incubated with MitoTracker Deep Red FM (200 μL, 25 nM) for an additional 15 min, and washed with DPBS twice. Then, the laser scanning confocal fluorescence images were captured. The green images of **3** or **5** were excited by 488 nm light, and the emission wavelength range was collected at 540±20 nm. The red images of mitochondria were excited by 633 nm light, and the emission wavelength range was collected at 665±20 nm. Colocalization was analyzed by ImageJ software.(Schneider et al., 2012)

Subcellular Localization of Lysosomes

Cells were incubated with DPBS dispersion of **3** or **5** (200 μL, 5 μg/mL) in CO_2 incubator for 30 min, and washed with DPBS twice. After additional 4 h incubation, cells were incubated with LysoTracker Red DND-99 (200 μL, 50 nM) for an additional 15 min, and washed with DPBS twice. Then, the laser scanning confocal fluorescence images were captured. The green images of **3** or **5** were excited by 488 nm light, and the emission wavelength range was collected at 540±20 nm. The orange images of lysosomes were excited by 561 nm light, and the

emission wavelength range was collected at 590±20 nm. Colocalization was analyzed by ImageJ software.(Schneider et al., 2012)

Mitochondrial Membrane Potential (MMP, ΔΨ)

Cells were incubated with DPBS dispersion of **3** or **5** (200 μL, 0.2 μM, BODIPY equiv) in CO_2 incubator for 30 min, and washed with DPBS twice. Then, the cells were exposed to green LED (40 mW/cm^2) for 4 min. The cells without green LED irradiation were used as control. After additional 4 h incubation, the cells were incubated with JC-1 (200 μL, 10 μg/mL) for 10 min, and washed with DPBS twice. Next, the laser scanning confocal fluorescence images were captured. The green images of monomer were excited by 488 nm light, and the emission wavelength range was collected at 530±15 nm. The red images of J-aggregate were excited by 514 nm light, and the emission wavelength range was collected at 590±17 nm. The mean fluorescence intensity (MFI) was analyzed by ImageJ software.(Schneider et al., 2012)

Lysosomal Membrane Permeabilization (LMP)

Cells were incubated with DPBS dispersion of **3** or **5** (200 μL, 0.2 μM, BODIPY equiv) in CO_2 incubator for 30 min, and washed with DPBS twice. Then, the cells were exposed to green LED (40 mW/cm^2) for 4 min. The cells without green LED irradiation were used as control. After additional 4 h incubation, the cells were incubated with AO (200 μL, 5 μg/mL) for 10 min, and washed with DPBS twice. Next, the laser scanning confocal fluorescence images were captured. The green images were excited by 488 nm light, and the emission wavelength range was collected at 530±20 nm. The red images were excited by 488 nm light, and the emission wavelength range was collected at 640±20 nm.

MCF-7 Xenograft Model

Animal experiments were reviewed and approved by the Ethics Committee of Shandong Normal University (Jinan, China). All methods were performed in accordance with the relevant guidelines and regulations on experimental animals.

Nude mice (BALB/c-nu♀, aged 5 weeks, 15~20 g) were purchased from the Beijing Vital River Laboratory Animal Technology Co., Ltd. MCF-7 cancer cells (5×10^6 cells) suspended in DPBS (100 μL) were subcutaneously injected into the flanks of each mice to establish MCF-7 xenograft model. Length (L) and width (W) of the tumor were determined by digital calipers. The tumor volume (V) was calculated by the formula: $V = 1/2 \times L \times W^2$. When the tumor size reached ~150 mm^3, animals were used in the experiments.

In Vivo PDT Experiment

The nude mice bearing MCF-7 tumors (n=30) were randomly distributed into 6 groups: (i) control group, DPBS injection (50 μL); (ii) **2**+laser group, DPBS dispersion of **2** injection (50 μL, 0.09 mg/mL, 150 μM, BODIPY equiv), green laser irradiation (1 W/cm^2, 10 min); (iii) **3** group, DPBS dispersion of **3** injection (50 μL, 1.1 mg/mL, 150 μM, BODIPY equiv); (iv) **3**+light group, DPBS dispersion of **3** injection (50 μL, 1.1 mg/mL, 150 μM, BODIPY equiv), green laser irradiation (1 W/cm^2, 10 min); (v) **5** group, DPBS dispersion of **5** injection (50 μL, 0.97 mg/mL, 150 μM, BODIPY equiv); (vi) **5**+light group, DPBS dispersion of **5** injection (50 μL, 0.97 mg/mL, 150 μM, BODIPY equiv), green laser irradiation (1 W/cm^2, 10 min). After intratumoral injection, the nude mice were feeding for 24 h, and for the treatment group, light treatment was performed on the tumor site. The mice continued to be fed for 14 days. The tumor volume and nude mouse body weight were recorded daily during the experimental period.

Histopathological Examination

At the end of the treatment, the nude mice were dissected, and major organs (heart, liver, spleen, lung, and kidney) were harvested and fixed in formalin fixative to make paraffin section for hematoxylin and eosin (H&E) staining.

Supplemental References

Bai, L., Phua, S.Z.F., Lim, W.Q., Jana, A., Luo, Z., Tham, H.P., Zhao, L., Gao, Q., and Zhao, Y. (2016). Nanoscale covalent organic frameworks as smart carriers for drug delivery. Chem Commun *52*, 4128-4131. DOI: 10.1039/C6CC00853D

Bhanja, P., Mishra, S., Manna, K., Mallick, A., Das Saha, K., and Bhaumik, A. (2017). Covalent Organic Framework Material Bearing Phloroglucinol Building Units as a Potent Anticancer Agent. ACS Appl Mater Interfaces *9*, 31411-31423. DOI: 10.1021/acsami.7b07343

Bunck, D.N., and Dichtel, W.R. (2013). Postsynthetic functionalization of 3D covalent organic frameworks. Chem Commun *49*, 2457-2459. DOI: 10.1039/C3CC40358K

Chandra, S., Kandambeth, S., Biswal, B.P., Lukose, B., Kunjir, S.M., Chaudhary, M., Babarao, R., Heine, T., and Banerjee, R. (2013). Chemically Stable Multilayered Covalent Organic Nanosheets from Covalent Organic Frameworks via Mechanical Delamination. J Am Chem Soc *135*, 17853-17861. DOI: 10.1021/ja408121p

Chen, L., Furukawa, K., Gao, J., Nagai, A., Nakamura, T., Dong, Y., and Jiang, D. (2014). Photoelectric Covalent Organic Frameworks: Converting Open Lattices into Ordered Donor–Acceptor Heterojunctions. J Am Chem Soc *136*, 9806-9809. DOI: 10.1021/ja502692w

Das, G., Benyettou, F., Sharama, S.K., Prakasam, T., Gándara, F., de la Peña-O'Shea, V.A., Saleh, N.i., Pasricha, R., Jagannathan, R., Olson, M.A., *et al.* (2018). Covalent organic nanosheets for bioimaging. Chem Sci *9*, 8382-8387. DOI: 10.1039/C8SC02842G

Daugherty, M.C., Vitaku, E., Li, R.L., Evans, A.M., Chavez, A., and Dichtel, W. (2019). Improved Synthesis of β-Ketoenamine-Linked Covalent Organic Frameworks via Monomer Exchange Reactions. Chem Commun *55*, 2680-2683. DOI: 10.1039/C8CC08957D

Dong, B., Wang, L., Zhao, S., Ge, R., Song, X., Wang, Y., and Gao, Y. (2016). Immobilization of ionic liquids to covalent organic frameworks for catalyzing the formylation of amines with CO2 and phenylsilane. Chem Commun *52*, 7082-7085. DOI: 10.1039/C6CC03058K

Fang, Q., Wang, J., Gu, S., Kaspar, R.B., Zhuang, Z., Zheng, J., Guo, H., Qiu, S., and Yan, Y. (2015). 3D Porous Crystalline Polyimide Covalent Organic Frameworks for Drug Delivery. J Am Chem Soc *137*, 8352-8355. DOI: 10.1021/jacs.5b04147

Feng, X., Ding, X., Chen, L., Wu, Y., Liu, L., Addicoat, M., Irle, S., Dong, Y., and Jiang, D. (2016). Two-dimensional artificial light-harvesting antennae with predesigned high-order structure and robust photosensitising activity. Sci Rep *6*, 32944. DOI: 10.1038/srep32944

Guan, Q., Zhou, L.-L., Li, Y.-A., and Dong, Y.-B. (2018). Diiodo-Bodipy-Encapsulated Nanoscale Metal–Organic Framework for pH-Driven Selective and Mitochondria Targeted Photodynamic Therapy. Inorg Chem *57*, 10137-10145. DOI: 10.1021/acs.inorgchem.8b01316

Guo, H., Wang, J., Fang, Q., Zhao, Y., Gu, S., Zheng, J., and Yan, Y. (2017). A quaternary-ammonium-functionalized

covalent organic framework for anion conduction. CrystEngComm *19*, 4905-4910. DOI: 10.1039/C7CE00042A

Guo, Z., Zou, Y., He, H., Rao, J., Ji, S., Cui, X., Ke, H., Deng, Y., Yang, H., Chen, C., *et al.* (2016). Bifunctional Platinated Nanoparticles for Photoinduced Tumor Ablation. Adv Mater *28*, 10155-10164. DOI: 10.1002/adma.201602738

Haase, F., Troschke, E., Savasci, G., Banerjee, T., Duppel, V., Dörfler, S., Grundei, M.M.J., Burow, A.M., Ochsenfeld, C., Kaskel, S., *et al.* (2018). Topochemical conversion of an imine- into a thiazole-linked covalent organic framework enabling real structure analysis. Nat Commun *9*, 2600. DOI: 10.1038/s41467-018-04979-y

Han, X., Huang, J., Yuan, C., Liu, Y., and Cui, Y. (2018). Chiral 3D Covalent Organic Frameworks for High Performance Liquid Chromatographic Enantioseparation. J Am Chem Soc *140*, 892-895. DOI: 10.1021/jacs.7b12110

Huang, L., Li, Z., Zhao, Y., Yang, J., Yang, Y., Pendharkar, A.I., Zhang, Y., Kelmar, S., Chen, L., Wu, W., *et al.* (2017). Enhancing Photodynamic Therapy through Resonance Energy Transfer Constructed Near-Infrared Photosensitized Nanoparticles. Adv Mater *29*, 1604789. DOI: 10.1002/adma.201604789

Huang, L., Li, Z., Zhao, Y., Zhang, Y., Wu, S., Zhao, J., and Han, G. (2016). Ultralow-Power Near Infrared Lamp Light Operable Targeted Organic Nanoparticle Photodynamic Therapy. J Am Chem Soc *138*, 14586-14591. DOI: 10.1021/jacs.6b05390

Huang, N., Chen, X., Krishna, R., and Jiang, D. (2015a). Two-Dimensional Covalent Organic Frameworks for Carbon Dioxide Capture through Channel-Wall Functionalization. Angew Chem Int Ed *54*, 2986-2990. DOI: 10.1002/anie.201411262

Huang, N., Krishna, R., and Jiang, D. (2015b). Tailor-Made Pore Surface Engineering in Covalent Organic Frameworks: Systematic Functionalization for Performance Screening. J Am Chem Soc *137*, 7079-7082. DOI: 10.1021/jacs.5b04300

Hynek, J., Zelenka, J., Rathouský, J., Kubát, P., Ruml, T., Demel, J., and Lang, K. (2018). Designing Porphyrinic Covalent Organic Frameworks for the Photodynamic Inactivation of Bacteria. ACS Appl Mater Interfaces *10*, 8527-8535. DOI: 10.1021/acsami.7b19835

Jiang, Z., Wang, Y., Sun, L., Yuan, B., Tian, Y., Xiang, L., Li, Y., Li, Y., Li, J., and Wu, A. (2019). Dual ATP and pH responsive ZIF-90 nanosystem with favorable biocompatibility and facile post-modification improves therapeutic outcomes of triple negative breast cancer in vivo. Biomaterials *197*, 41-50. DOI: 10.1016/j.biomaterials.2019.01.001

Kandambeth, S., Mallick, A., Lukose, B., Mane, M.V., Heine, T., and Banerjee, R. (2012). Construction of Crystalline 2D Covalent Organic Frameworks with Remarkable Chemical (Acid/Base) Stability via a Combined Reversible and Irreversible Route. J Am Chem Soc *134*, 19524-19527. DOI: 10.1021/ja308278w

Li, W., Yang, C.-X., and Yan, X.-P. (2017). A versatile covalent organic framework-based platform for sensing biomolecules. Chem Commun *53*, 11469-11471. DOI: 10.1039/C7CC06244C

Li, X., Zhang, C., Cai, S., Lei, X., Altoe, V., Hong, F., Urban, J.J., Ciston, J., Chan, E.M., and Liu, Y. (2018). Facile transformation of imine covalent organic frameworks into ultrastable crystalline porous aromatic frameworks. Nat Commun *9*, 2998. DOI: 10.1038/s41467-018-05462-4

Li, Z., Ding, X., Feng, Y., Feng, W., and Han, B.-H. (2019). Structural and Dimensional Transformations between Covalent Organic Frameworks via Linker Exchange. Macromolecules *52*, 1257-1265. DOI: 10.1021/acs.macromol.8b01814

Lin, G., Ding, H., Chen, R., Peng, Z., Wang, B., and Wang, C. (2017). 3D Porphyrin-Based Covalent Organic Frameworks. J Am Chem Soc *139*, 8705-8709. DOI: 10.1021/jacs.7b04141

Liu, L., Fu, L., Jing, T., Ruan, Z., and Yan, L. (2016). pH-Triggered Polypeptides Nanoparticles for Efficient BODIPY Imaging-Guided Near Infrared Photodynamic Therapy. ACS Appl Mater Interfaces *8*, 8980-8990. DOI: 10.1021/acsami.6b01320

Liu, S., Hu, C., Liu, Y., Zhao, X., Pang, M., and Lin, J. (2019a). One-pot synthesis of DOX@covalent organic framework with enhanced chemotherapeutic efficacy. Chem Eur J. DOI: 10.1002/chem.201806242

Liu, T., Hu, X., Wang, Y., Meng, L., Zhou, Y., Zhang, J., Chen, M., and Zhang, X. (2017a). Triazine-based covalent organic frameworks for photodynamic inactivation of bacteria as type-II photosensitizers. J Photochem Photobiol, B *175*, 156-162. DOI: 10.1016/j.jphotobiol.2017.07.013

Liu, W., Cao, Y., Wang, W., Gong, D., Cao, T., Qian, J., Iqbal, K., Qin, W., and Guo, H. (2019b). Mechanochromic luminescent covalent organic frameworks for highly selective hydroxyl radical detection. Chem Commun *55*, 167-170. DOI: 10.1039/C8CC07783E

Liu, Y., Li, Z., Chen, L., and Xie, Z. (2017b). Near infrared BODIPY-Platinum conjugates for imaging, photodynamic therapy and chemotherapy. Dyes Pigm *141*, 5-12. DOI: 10.1016/j.dyepig.2017.01.075

Lohse, M.S., Stassin, T., Naudin, G., Wuttke, S., Ameloot, R., De Vos, D., Medina, D.D., and Bein, T. (2016). Sequential Pore Wall Modification in a Covalent Organic Framework for Application in Lactic Acid Adsorption. Chem Mater *28*, 626-631. DOI: 10.1021/acs.chemmater.5b04388

Lu, Q., Ma, Y., Li, H., Guan, X., Yusran, Y., Xue, M., Fang, Q., Yan, Y., Qiu, S., and Valtchev, V. (2018). Postsynthetic Functionalization of Three‐Dimensional Covalent Organic Frameworks for Selective Extraction of Lanthanide Ions. Angew Chem Int Ed *57*, 6042-6048. DOI: 10.1002/anie.201712246

Mitra, S., Kandambeth, S., Biswal, B.P., Khayum M., A., Choudhury, C.K., Mehta, M., Kaur, G., Banerjee, S., Prabhune, A., Verma, S., *et al*. (2016). Self-Exfoliated Guanidinium-Based Ionic Covalent Organic Nanosheets (iCONs). J Am Chem Soc *138*, 2823-2828. DOI: 10.1021/jacs.5b13533

Mitra, S., Sasmal, H.S., Kundu, T., Kandambeth, S., Illath, K., Díaz Díaz, D., and Banerjee, R. (2017). Targeted Drug Delivery in Covalent Organic Nanosheets (CONs) via Sequential Postsynthetic Modification. J Am Chem Soc *139*, 4513-4520. DOI: 10.1021/jacs.7b00925

Nagai, A., Chen, X., Feng, X., Ding, X., Guo, Z., and Jiang, D. (2013). A Squaraine‐Linked Mesoporous Covalent Organic Framework. Angew Chem Int Ed *52*, 3770-3774. DOI: 10.1002/anie.201300256

Nagai, A., Guo, Z., Feng, X., Jin, S., Chen, X., Ding, X., and Jiang, D. (2011). Pore surface engineering in covalent organic frameworks. Nat Commun *2*, 536. DOI: 10.1038/ncomms1542

Qian, C., Qi, Q.-Y., Jiang, G.-F., Cui, F.-Z., Tian, Y., and Zhao, X. (2017). Toward Covalent Organic Frameworks Bearing Three Different Kinds of Pores: The Strategy for Construction and COF-to-COF Transformation via Heterogeneous Linker Exchange. J Am Chem Soc *139*, 6736-6743. DOI: 10.1021/jacs.7b02303

Qian, H.-L., Li, Y., and Yan, X.-P. (2018). A building block exchange strategy for the rational fabrication of de novo unreachable amino-functionalized imine-linked covalent organic frameworks. J Mater Chem A *6*, 17307-17311. DOI: 10.1039/C8TA06195E

Rager, S., Dogru, M., Werner, V., Gavryushin, A., Gotz, M., Engelke, H., Medina, D.D., Knochel, P., and Bein, T. (2017). Pore wall fluorescence labeling of covalent organic frameworks. CrystEngComm *19*, 4886-4891. DOI: 10.1039/C7CE00684E

Ruyra, À., Yazdi, A., Espín, J., Carné-Sánchez, A., Roher, N., Lorenzo, J., Imaz, I., and Maspoch, D. (2015). Synthesis, Culture Medium Stability, and In Vitro and In Vivo Zebrafish Embryo Toxicity of Metal–Organic Framework Nanoparticles. Chem Eur J *21*, 2508-2518. DOI: 10.1002/chem.201405380

Schneider, C.A., Rasband, W.S., and Eliceiri, K.W. (2012). NIH Image to ImageJ: 25 years of image analysis. Nat Methods *9*, 671-675. DOI: 10.1038/nmeth.2089

Sun, Q., Aguila, B., Earl, L.D., Abney, C.W., Wojtas, L., Thallapally, P.K., and Ma, S. (2018a). Covalent Organic Frameworks as a Decorating Platform for Utilization and Affinity Enhancement of Chelating Sites for Radionuclide Sequestration. Adv Mater *30*, 1705479. DOI: 10.1002/adma.201705479

Sun, Q., Aguila, B., Perman, J., Earl, L.D., Abney, C.W., Cheng, Y., Wei, H., Nguyen, N., Wojtas, L., and Ma, S. (2017). Postsynthetically Modified Covalent Organic Frameworks for Efficient and Effective Mercury Removal. J Am Chem Soc *139*, 2786-2793. DOI: 10.1021/jacs.6b12885

Sun, Q., Aguila, B., Perman, J.A., Butts, T., Xiao, F.-S., and Ma, S. (2018b). Integrating Superwettability within Covalent Organic Frameworks for Functional Coating. Chem *4*, 1726-1739. DOI: 10.1016/j.chempr.2018.05.020

Tamames-Tabar, C., Cunha, D., Imbuluzqueta, E., Ragon, F., Serre, C., Blanco-Prieto, M.J., and Horcajada, P. (2014). Cytotoxicity of nanoscaled metal–organic frameworks. J Mater Chem B *2*, 262-271. DOI: 10.1039/C3TB20832J

Tan, J., Namuangruk, S., Kong, W., Kungwan, N., Guo, J., and Wang, C. (2016). Manipulation of Amorphous-to-Crystalline Transformation: Towards the Construction of Covalent Organic Framework Hybrid Microspheres with NIR Photothermal Conversion Ability. Angew Chem Int Ed *55*, 13979-13984. DOI: 10.1002/anie.201606155

van Meerloo, J., Kaspers, G.J.L., and Cloos, J. (2011). Cell Sensitivity Assays: The MTT Assay. In Cancer Cell Culture: Methods and Protocols, I.A. Cree, ed. (New Jersey: Humana Press), pp. 237-245. DOI: 10.1007/978-1-61779-080-5_20

Vardhan, H., Verma, G., Ramani, S., Nafady, A., Al-Enizi, A.M., Pan, Y., Yang, Z., Yang, H., and Ma, S. (2019). Covalent Organic Framework Decorated with Vanadium as a New Platform for Prins Reaction and Sulfide Oxidation. ACS Appl Mater Interfaces *11*, 3070-3079. DOI: 10.1021/acsami.8b19352

Vyas, V.S., Vishwakarma, M., Moudrakovski, I., Haase, F., Savasci, G., Ochsenfeld, C., Spatz, J.P., and Lotsch, B.V. (2016). Exploiting Noncovalent Interactions in an Imine-Based Covalent Organic Framework for Quercetin Delivery. Adv Mater *28*, 8749-8754. DOI: 10.1002/adma.201603006

Waller, P.J., AlFaraj, Y.S., Diercks, C.S., Jarenwattananon, N.N., and Yaghi, O.M. (2018). Conversion of Imine to Oxazole and Thiazole Linkages in Covalent Organic Frameworks. J Am Chem Soc *140*, 9099-9103. DOI: 10.1021/jacs.8b05830

Waller, P.J., Lyle, S.J., Osborn Popp, T.M., Diercks, C.S., Reimer, J.A., and Yaghi, O.M. (2016). Chemical Conversion of Linkages in Covalent Organic Frameworks. J Am Chem Soc *138*, 15519-15522. DOI: 10.1021/jacs.6b08377

Wang, P., Zhou, F., Zhang, C., Yin, S.-Y., Teng, L., Chen, L., Hu, X.-X., Liu, H.-W., Yin, X., and Zhang, X.-B. (2018). Ultrathin two-dimensional covalent organic framework nanoprobe for interference-resistant two-photon fluorescence bioimaging. Chem Sci *9*, 8402-8408. DOI: 10.1039/C8SC03393E

Wang, W., Wang, L., Li, Z., and Xie, Z. (2016). BODIPY-containing nanoscale metal-organic frameworks for photodynamic therapy. Chem Commun *52*, 5402-5405. DOI: 10.1039/C6CC01048B

Xu, F., Xu, H., Chen, X., Wu, D., Wu, Y., Liu, H., Gu, C., Fu, R., and Jiang, D. (2015a). Radical Covalent Organic Frameworks: A General Strategy to Immobilize Open-Accessible Polyradicals for High-Performance Capacitive Energy Storage. Angew Chem Int Ed *54*, 6814-6818. DOI: 10.1002/anie.201501706

Xu, H., Chen, X., Gao, J., Lin, J., Addicoat, M., Irle, S., and Jiang, D. (2014). Catalytic covalent organic frameworks via pore surface engineering. Chem Commun *50*, 1292-1294. DOI: 10.1039/C3CC48813F

Xu, H., Gao, J., and Jiang, D. (2015b). Stable, crystalline, porous, covalent organic frameworks as a platform for chiral organocatalysts. Nat Chem *7*, 905-912. DOI: 10.1038/nchem.2352

Xue, F., Wei, P., Ge, X., Zhong, Y., Cao, C., Yu, D., and Yi, T. (2018). A pH-responsive organic photosensitizer specifically activated by cancer lysosomes. Dyes Pigm *156*, 285-290. DOI: 10.1016/j.dyepig.2018.04.008

Zhang, G., Li, X., Liao, Q., Liu, Y., Xi, K., Huang, W., and Jia, X. (2018). Water-dispersible PEG-curcumin/amine-functionalized covalent organic framework nanocomposites as smart carriers for in vivo drug delivery. Nat Commun *9*, 2785. DOI: 10.1038/s41467-018-04910-5

Zheng, H., Zhang, Y., Liu, L., Wan, W., Guo, P., Nyström, A.M., and Zou, X. (2016). One-pot Synthesis of Metal–Organic Frameworks with Encapsulated Target Molecules and Their Applications for Controlled Drug Delivery. J Am Chem Soc *138*, 962-968. DOI: 10.1021/jacs.5b11720

Zhou, L.-L., Guan, Q., Li, Y.-A., Zhou, Y., Xin, Y.-B., and Dong, Y.-B. (2018). One-Pot Synthetic Approach toward Porphyrinatozinc and Heavy-Atom Involved Zr-NMOF and Its Application in Photodynamic Therapy. Inorg Chem *57*, 3169-3176. DOI: 10.1021/acs.inorgchem.7b03204
